# ALKBH5 activates FAK signaling through m6A demethylation in *ITGB1* mRNA and enhances tumor-associated lymphangiogenesis and lymph node metastasis in ovarian cancer

**DOI:** 10.7150/thno.77441

**Published:** 2023-01-05

**Authors:** Rui Sun, Lin Yuan, Yi Jiang, Yicong Wan, Xiaolling Ma, Jing Yang, Guodong Sun, Shulin Zhou, Hui Wang, Jiangnan Qiu, Lin Zhang, Wenjun Cheng

**Affiliations:** Department of Gynecology, the First Affiliated Hospital of Nanjing Medical University, Nanjing 210029, Jiangsu, China

**Keywords:** lymphatic metastasis, epithelial ovarian cancer, N6-methyladenosine, ALKBH5, ITGB1

## Abstract

**Background:** Lymph node (LN) metastasis is common in patients with epithelial ovarian cancer (EOC) and is associated with poor prognosis. Tumor-associated lymphangiogenesis is the first stage of LN metastasis. Research on lymphangiogenesis and lymph node metastases can help develop new anti-LN-targeted therapies. Aberrant N6-methyladenosine (m6A) modifications have been reported to be linked to LN metastasis in several cancers, however, their role in EOC lymphangiogenesis and LN metastasis remains unclear.

**Methods:** m6A levels in EOC tissues with or without LN metastases were evaluated by dot blot analysis. Real-time polymerase chain reaction (PCR) and immunofluorescence were used to examine the expression of m6A-related enzymes. Additionally, *in vitro* and *in vivo* functional studies were performed to discover the importance of the AlkB homolog 5 (*ALKBH5*) gene in EOC lymphatic metastasis. To identify the downstream target genes regulated by ALKBH5, we performed RNA pulldown, RNA-binding protein immunoprecipitation-quantitative PCR, co-immunoprecipitation, m6A-modified RNA immunoprecipitation-quantitative PCR, and luciferase reporter assays.

**Results:** m6A modification was reduced in ovarian cancers with LN metastases. ALKBH5 overexpression increased tumor-associated lymphangiogenesis and LN metastasis both *in vitro* and *in vivo*. ALKBH5 overexpression also reversed the m6A modification in *ITGB1* mRNA and suppressed the YTHDF2 protein-mediated m6A-dependent *ITGB1* mRNA degradation, which resulted in increased expression of ITGB1 and phosphorylation of the focal adhesion kinase (FAK) and Src proto-oncogene proteins, thereby increasing LN metastasis. Furthermore, hypoxia induced the expression of hypoxia inducible factor 1 subunit alpha, which increased ALKBH5 expression and enhanced LN metastasis in EOC.

**Conclusions:** The ALKBH5/m6A-ITGB1/FAK signalling axis is important in ovarian cancer lymphangiogenesis and LN metastasis. Antibodies that block ITGB1 and FAK kinase-inhibitors are promising anti-metastatic agents.

## Introduction

Epithelial ovarian cancer (EOC) is the most lethal gynaecological malignancy 1. Lymph node (LN) metastasis occurs in 44-53% of advanced EOC and 13.5% of early-stage EOC 2-5. Although EOC typically spreads via intraperitoneal dissemination, tumor cells are more likely to invade to other organs via the lymphatic system 6,7. Importantly, LN metastasis is an independent risk factor for EOC treatment resistance, which results in reduced patient survival 8,9. However, the molecular mechanisms underlying LN metastasis in EOC remain unclear.

Tumor lymphangiogenesis is the first stage of LN metastasis, followed by tumor cell dissemination to the lymphatic vessels, tumor cell transfer to LNs through the lymphatic system, cell seeding, colonisation and growth in LNs 10-14. Vascular endothelial growth factor (VEGF-C) and VEGF-D as well as their cognate receptor VEGF-R3 have been reported to be crucial for the onset of lymphangiogenesis. However, no good therapy has been in the clinic that blocks lymphangiogeneisis and lymphatic metastasis. Therefore, exploring the regulatory mechanisms of lymphangiogenesis and LN metastasis in EOC may improve the efficacy of anti-LN targets.

The most prevalent internal modification in eukaryotic mRNA is N6-methyladenosine (m6A) methylation, which occurs at the N6-position of adenosine and is regulated by methyltransferases (writers), demethylases (erasers), and recognition proteins (readers) 14. RNA processing, translation, and degradation are affected by abnormal m6A modification, which has been implicated in tumor progression 15. Recent studies have revealed a link between m6A-related gene expression and tumor lymphatic metastasis 16,17. Methyltransferase 3 (METTL3) stimulates lymphangiogenesis by modulating VEGF-C in mice 18. Insulin-like growth factor 2 mRNA binding protein 2 (IGF2BP2) can promote LN metastasis in head and neck squamous cell carcinoma 19. However, it is unclear whether the aberrant m6A regulatory mechanism could influence LN metastasis, especially early lymphangiogenesis in ovarian cancer.

In this study, we have found that AlkB homolog 5 (ALKBH5) is associated with LN metastasis in ovarian cancer. ALKBH5 promotes tumor-associated lymphangiogenesis and then LN metastasis, in which ALKBH5 stimulates downstream focal adhesion kinase (FAK)/Src proto-oncogene (Src) signalling and boosts integrin subunit beta 1 (ITGB1) expression by disrupting the YTHDF2 protein-mediated m6A degradation pathway.

## Methods

### Patient samples

Tumor tissues were acquired from patients diagnosed as ovarian cancer and underwent surgery between January 2012 and January 2018 in our hospital. Patients who did not receive systematic LN dissection were excluded. The specimens were immediately frozen in liquid nitrogen until analysis. The characteristics of the 192 patients were listed in **Table S1**. Written informed consent and Institutional ethics approval were obtained (No. 2018-SRFA-052).

### Cell lines and culture conditions

EOC cell lines HO8910, A2780, and human lymphatic endothelial cell (HLEC) were cultured in RPMI1640 (Gibco, Grand Island, NY, USA), supplemented with 10% foetal bovine serum (Gibco) and 1% penicillin/streptomycin, at 37 ℃. The cells were regularly tested for mycoplasma contamination.

### RNA extraction and quantitative reverse transcription-PCR

An animal RNA isolation kit (Beyotime, Shanghai, China) was used to extract total RNA from the cells and tissues, and cDNA was generated using a reverse transcription kit (Vazyme, Nanjing, China) according to the manufacturer's instructions. mRNA expression was detected using a SYBR Green PCR Kit (Vazyme). **Table S2** listed the sequences of the primers used in PCR.

### Western blot analysis and protein extraction

Total proteins from cells and tissues were lysed in RIPA buffer containing protease inhibitors (Beyotime), and protein concentrations were determined using the BCA method (Beyotime). Proteins were denatured, and their expression levels were evaluated using western blotting. The antibodies used for western blotting are listed in **Table S3**.

### Lentivirus infection and siRNA transfection

Lentiviral vectors encoding full-length ALKBH5 and control sequences were purchased from Genechem (Shanghai, China). For knockdown, short hairpin RNA of human ALKBH5 and control cloned into a green fluorescent protein-puromycin lentiviral vector was constructed by WZ biosciences (Shandong, China). Cells were incubated with lentivirus and polybrene for 24 h, and 5 μg/mL puromycin (Sigma-Aldrich, St. Louis, MO, USA) was used to screen for cells stably expressing ALKBH5. Small interfering RNA (siRNA) sequences were transfected into cells using Lipofectamine 3000 (Invitrogen, Carlsbad, CA, USA). siRNA was synthesized by Tsingke (Nanjing, China). The target sequences of the genes are shown in **Table S4**.

### Dot blot assay

An mRNA purification kit was used to isolate and purify mRNA from total RNA (Beyotime). All procedures were performed according to the kit instructions. After quantification and denaturation (95 °C, 5 min), mRNA was loaded onto Amersham HyBond N^+^ membranes (Amersham, UK). UV irradiation was used to cross-link the mRNA to the membrane twice. As a control, photographs were taken after incubating methylene blue with the mRNA. The membranes were rinsed with phosphate-buffered saline (PBS), containing 0.1% Tween 20, and blocked with 5% non-fat milk, followed by overnight incubation with an anti-m6A antibody at 4 °C and recognition with a horseradish peroxidase-conjugated secondary antibody. The antibodies used in the western blot assay are listed in **Table S3**.

### Immunohistochemical and immunofluorescence assays

Surgical samples were fixed in a 10% formaldehyde solution. Embedding, sectioning, deparaffinization, hydration, antigen retrieval, serum blocking, and primary antibody incubation at 4 °C overnight and incubation with horseradish peroxidase or fluorescent-labelled secondary antibodies were performed as described previously 19,20. DAPI was used to counter-stain the nuclei. A Zeiss microscope (Oberkochen, Germany) was used to obtain the images.

### Transwell migration and invasion assay

Cell migration and invasion abilities were detected using a transwell system equipped with 8-µm-pore polycarbonate filters (Corning, Inc., Corning, NY, USA). The upper chamber was seeded with 2 × 10^5^ cells in serum-free media and coated with or without Matrigel (Corning, Inc.). RPMI1640 containing 10% foetal bovine serum was added to the lower chamber. After 24 h, the migratory or invaded cells were fixed, stained, and counted under a light microscope.

### HLEC tube formation assay

A2780 and H08910 cells were incubated for 24 h in a fresh medium containing 1% foetal bovine serum. The conditioned medium was collected and centrifuged for 10 min at 800 rpm to remove cells and debris. For subsequent tube formation experiments, 5 × 10^5^ HLECs were grown in a mixed medium, composed of concentrated conditioned media obtained from EOC cells, on Matrigel-coated 96-well plates (Corning, Inc.). The potential of HLECs to form tubes was evaluated after 16 h of incubation.

### Popliteal lymphatic metastasis model

A popliteal LN metastasis model was established to evaluate the metastasis ability of cancer cells 20,21. BALB/c nude mice (4-5 weeks old, 16-18 g) were purchased from the Experimental Animal Center, Nanjing Medical University (Nanjing, China). The animal study was approved by the Animal Research Ethics Committee of Nanjing Medical University (No. IACUC-2011035). We injected 8 × 10^5^ HO8910 cells into the footpads of mice. The mice were randomized two weeks after tumor cell injection to receive either an ITGB1 blocking antibody (Santa, CA, USA) or an IgG isotype antibody twice per week for four weeks 22,23. Y15 (30 mg/kg) against p-FAK (Tyr397) and PBS as a control were intraperitoneally injected twice per week for four weeks in the FAK-treatment assays. The main tumors and popliteal LNs were harvested and fixed in paraformaldehyde after six weeks of tumor cell inoculation. LN volumes were calculated as follows: LN volume (mm^3^) = (length [mm]) × (width [mm])^2^ × 0.52. The formalin-fixed, paraffin-embedded samples were analysed using immunohistochemistry and haematoxylin and eosin (H&E) staining.

### Co-immunoprecipitation

To obtain the supernatant of EOC, 1 × 10^7^ cells were washed twice with ice-cold PBS before being lysed with an IP lysis buffer (Beyotime) and then being centrifuged. The immunoprecipitation complex was formed by incubating the remaining 10% of the supernatant with IgG or IP antibodies (**Table S3**) for 2 h at 4°C. Pre-treated protein A/G magnetic beads (40 μL; Invitrogen) were added to each tube and incubated at 4 °C for 2 h. The protein and bead mixture was rinsed thrice with a high-salt or low-salt buffer before boiling it in a sodium dodecyl sulphate-polyacrylamide gel electrophoresis sample loading buffer (Beyotime) for 5 min.

### RNA-pulldown assay

The RNA-pulldown experiment was carried out as described in our previous study 25. Briefly, 3′-biotin RNA probes were synthesized by Tsingke. One m6A base, one adenine base, or one guanine base was mutated in the RNA probes. We used Lipofectamine 3000 (Invitrogen) to transfect the RNA probes into cells; after 24 h, the cells were lysed in an immunoprecipitation lysis buffer and the supernatants were obtained via centrifugation. The probe and protein complexes were enriched with streptomycin avidin C1 magnetic beads (Invitrogen), and the protein was denatured for western blot detection. The RNA probe sequences for RNA pulldown are shown in **Table S5**.

### RNA binding protein immunoprecipitation-quantitative PCR

A MagnaRIP RNA-Binding Protein Immunoprecipitation Kit (Millipore, Billerica, MA, USA) was used for the RNA immunoprecipitation (RIP) experiment. The cell lysates were treated with beads coated with 5 g antibodies overnight at 4 °C with rotation. The beads were rinsed five times with a high- or low-salt buffer after immunoprecipitation. A proteinase K digestion buffer was used to wash and elute the RNA-protein-magnetic bead complexes. Immunoprecipitated RNA was obtained through Trizol-chloroform RNA extraction. Finally, quantitative PCR (qPCR) was performed to detect enriched RNA, which was then normalized to the input. The antibodies used in the RIP assay are listed in **Table S3**.

### Methylated RNA immunoprecipitation (MeRIP)-qPCR

mRNA was isolated from total RNA samples using the methods previously described. The Magna MeRIP^TM^ m6A kit (Millipore) was used for MeRIP studies, and all procedures were performed according to the instructions. Briefly, 5 μg mRNA fragments, in the 100-200 nt range, were incubated with an IgG or m6A antibody for 2 h at 4 °C. Protein A magnetic beads were added, and the sample was incubated for another 2 h. A magnet was used to separate the beads, followed by five washes with a wash buffer, antibody digestion with proteinase K to yield the RNA, and recovery of the RNA fragments using phenol-chloroform RNA extraction. The m6A levels in *ITGB1* mRNA were analysed using qPCR. The specific primers are shown in **Table S2**.

### Chromatin immunoprecipitation-qPCR

Chromatin immunoprecipitation (ChIP) experiments were performed using an EZMagna ChIP G kit (Millipore) according to the manufacturer's instructions. The cells were washed twice with 1× PBS after cross-linking with 1% formaldehyde and quenching with glycine. After lysing the cells in lysis buffer, the cross-linked DNA was fragmented to 500 bp via sonication. The samples were centrifuged to obtain the supernatant, and 10% of the supernatant was retained as input. The remaining solution was divided in half and incubated overnight at 4 °C, after which the HIF-1α antibody and IgG antibody-magnetic bead complex were added. The immunoprecipitated complexes were separated using a magnetic stand, the magnetic beads were rinsed with high- and low-salt buffers, and the DNA fragments were digested with proteinase K to remove the antibody and purified. qPCR was used to assess DNA enrichment. The ChIP-qPCR primers are listed in **Table S2**.

### RNA stability assay

RNA stability was measured as described previously 24. Actinomycin D (5 μg/mL) was added to the EOC cells (Sigma). The cells were harvested at specific time points, and the total RNA was collected and reverse-transcribed into cDNA. The intracellular RNA levels were determined using qPCR, and the RNA degradation rate was calculated as described by Huang et al. 24.

### Protein stability assay

The EOC cells were cultured in 6-well plates. Cycloheximide (Sigma) was added to the medium, at a final concentration of 100 μg/mL, after the indicated treatments. At specific time points, total protein was extracted, and ITGB1 protein expression was detected using western blotting.

### Luciferase reporter assay

Luciferase reporter plasmids containing wild-type or mutant sequences of ITGB1 3′ untranslated region (UTR) were constructed by Tsingke. The wild-type or mutant plasmids were transfected into cells using Lipo3000 reagent (Invitrogen), and luciferase assays were performed using the Dual-Luciferase Reporter Assay Kit (Promega, Madison, WI, USA) according to the kit instructions. The plasmid sequences are shown in **Table S6**.

### Statistical analysis

All data are presented as the mean and standard deviation. Student's *t*-test was used to examine differences between groups. SPSS 16 software (SPSS, Inc., Chicago, IL, USA) was used for statistical analysis, and GraphPad Prism 5 software was used to prepare graphical presentations. Correlations were examined using Pearson's correlation coefficient analysis. Statistical significance was defined as *P* < 0.05.

## Results

### m6A modification was reduced in metastatic ovarian cancer and correlated with a high ALKBH5 expression

**Figure 1:**
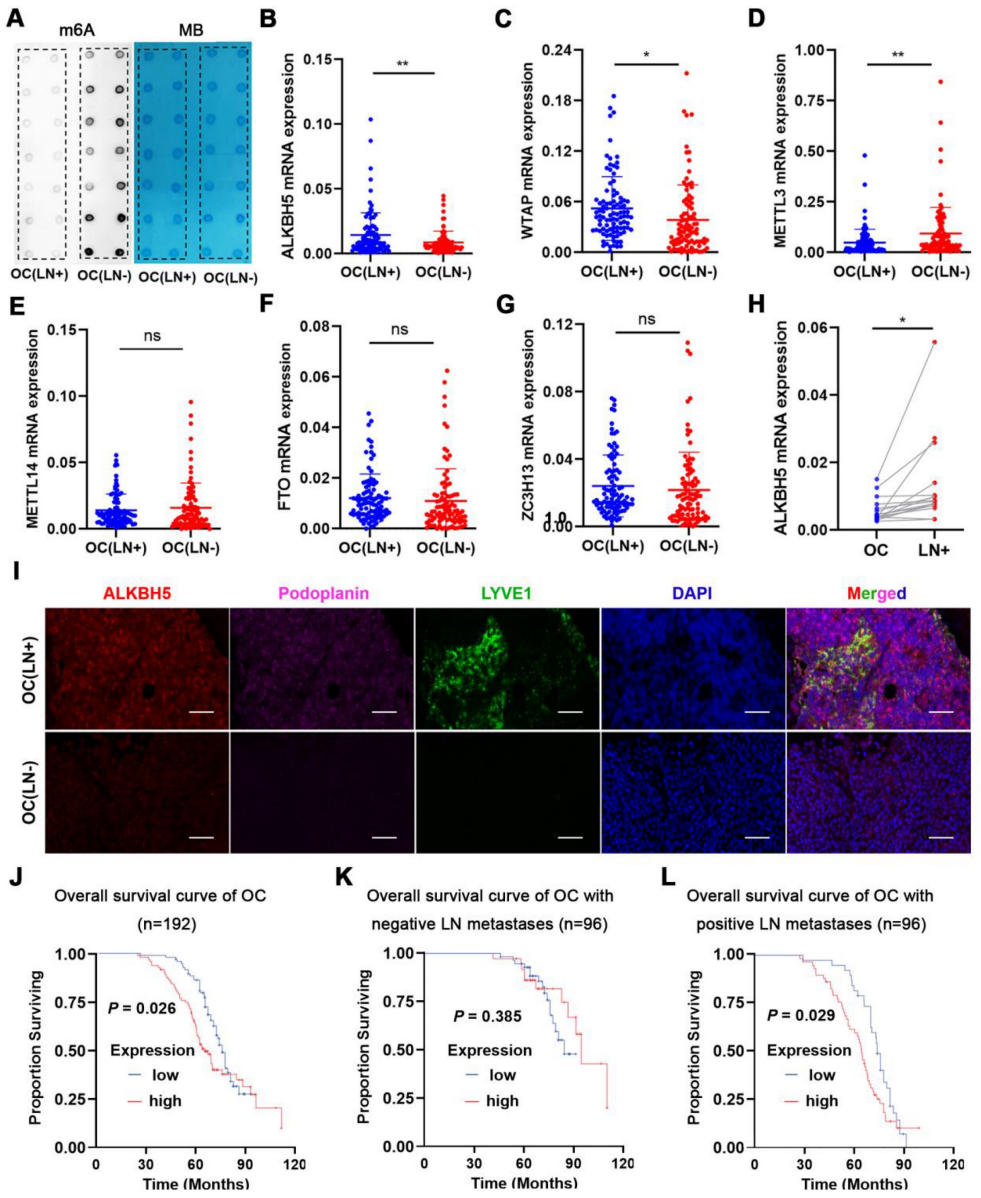
** ALKBH5 is associated with lymph node (LN) metastasis in ovarian cancer (OC).** (A) Dot blot analysis of m6A levels in OC tissues with or without LN metastasis. (B-G) qPCR analysis of (B) *ALKBH5*, (C) *WTAP*, (D) *METTL3*, (E) *METTL14*, (F) *FTO*, and (G) *ZC3H13* mRNA expression in OC tissues with or without LN metastasis (n = 192 cases). (H) qPCR analysis of *ALKBH5* mRNA expression in primary tumors and LN metastases in the same patient (n = 16 pairs). (I) Immunofluorescence staining of ALKBH5 and microlymphatic vessel density in OC tissues with and without LN metastasis. ALKBH5 expression levels were quantified using immunofluorescence (red), and microlymphatic vessel density was quantified via immunofluorescence using both the anti-LYVE-1 antibody (green) and the anti-podoplanin antibody (violet). Scale bars: 200 μm. (J) Kaplan-Meier analysis of overall survival in OC according to ALKBH5 expression (n = 192 cases). High and low expression of ALKBH5 was defined according to the median. (K) Overall survival of patients with OC without LN metastasis relative to ALKBH5 expression in our cohort (n = 96 cases). (L) Overall survival of patients with OC with LN metastasis relative to ALKBH5 expression in our cohort (n = 96 cases); error bars indicate the SD of the mean. Statistical significance was assessed using a two-tailed Student's *t* test. *P < 0.05, **P < 0.01, ns denotes not significant.

We measured m6A modifications in EOC tissues with or without LN metastasis, and it turned out that m6A modifications were considerably reduced in LN metastases (**Figure 1A**). Since m6A modifications were generated by methyltransferase and removed by demethyltransferase in eukaryotic RNA, we examined whether m6A modifications in ovarian cancer could be reduced by over-expression of demethyltransferase or knock-down of methyltransferase. The expression of m6A-related methyltransferases and demethyltransferases was evaluated in EOC tissues. *ALKBH5* and *WTAP* mRNA expression was increased in EOC tissues with LN metastases (**Figure 1B-C**), whereas METTL3 expression was reduced (**Figure 1D**). There were no significant differences in *METTL14*, *FTO*, and *ZC3H13* mRNA levels between the two groups (**Figure 1E-G**). These findings suggested that overexpression of ALKBH5 or low expression of METTL3 was the main cause of m6A downregulation. We further assessed the expression of ALKBH5 and METTL3 in the primary tumors and LN metastatic tissues. ALKBH5 expression was considerably high in the metastatic LNs (**Figure 1H**), whereas METTL3 expression did not significantly differ (**Figure S1A**). Thus, we hypothesised that demethylation was closely linked to reduced m6A modifications in EOC.

LYVE-1 and podoplanin were the most specific markers of lymphatic endothelium to distinguish lymphatic and blood vessels. Immunofluorescence staining revealed increased lymphatic vessel density (LYVE-1 and podoplanin marked) and ALKBH5 expression in EOC with LN metastasis (**Figure 1I**). Additionally, the ALKBH5 protein was highly expressed in EOC tissues with LN metastases (**Figure 1J**), which suggest that ALKBH5 played a unique role in EOC-mediated LN metastasis. According to univariate analysis, patients with high ALKBH5 expression had more advanced FIGO stages (*P* = 0.035), higher grade tumors (*P* = 0.008), and more frequent LN metastases (*P* = 0.004) than those with low ALKBH5 expression. There were no significant differences in the age (*P* = 0.134), histological type (*P* = 0.082), tumor size (*P* = 0.191), peritoneal cytology (*P* = 0.559), or complications of diabetes (*P* = 0.209) between the groups (**Table S1**). Based on these prognostic parameters, we assessed the relationship between ALKBH5 expression and the clinical outcomes in 192 EOC patients in our cohort. Kaplan-Meier analysis showed that high *ALKBH5* mRNA expression was associated with reduced OS in EOC. Patients with high or low ALKBH5 expression had median OS of 37.9 and 44 months, respectively. Although there were no significant variations in the OS duration among the 96 patients without LN metastasis (*P* = 0.385, **Figure 1K**), high ALKBH5 expression was a poor prognostic indicator in the 96 patients with LN metastasis (*P* = 0.029, **Figure 1L**). Furthermore, a dataset from The Cancer Genome Atlas (TCGA) was also proved that patients with elevated ALKBH5 expression had low OS and progression-free survival rates (**Figure S1B-C**).

### ALKBH5 promotes lymphangiogenesis and LN metastasis* in vivo* and* in vitro*

**Figure 2:**
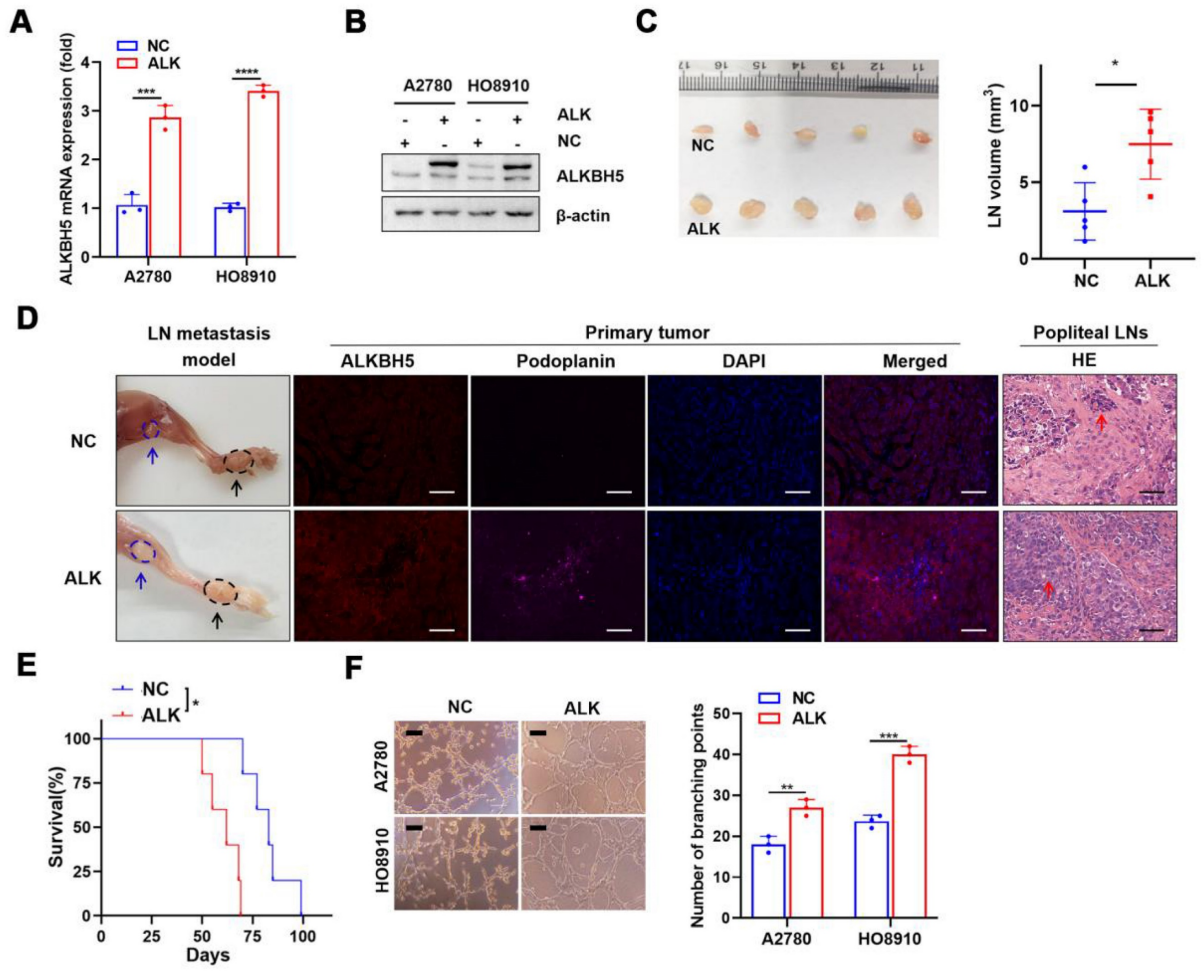
** ALKBH5 overexpression promotes lymphangiogenesis and lymph node (LN) metastasis *in vivo* and* in vitro*.** (A and B) qPCR (A) and western blot (B) analysis of the transfection efficiency of ALKBH5-overexpressing lentivirus in A2780 and HO8910. (C) Representative images (left) and volume quantification (right) of enucleated popliteal LNs inoculated with the indicated cells (n = 5 per group). (D) Representative images: nude mouse model of popliteal LN metastasis, different ALKBH5 expression levels and microlymphatic vessel density in the primary tumors, haematoxylin and eosin (H&E) staining of the popliteal LNs. HO8910 cells were injected into the footpads of nude mice, and primary tumors and popliteal LNs were enucleated and analysed. Blue arrow: popliteal LN. Black arrow: primary tumor in footpad; ALKBH5 expression levels and microlymphatic vessel density quantified using Immunofluorescence with the anti-ALKBH5 antibody and the anti-podoplanin antibody. H&E staining of the LN status indicating tumor cell infiltration (red arrow). Two representative cases are shown. (E) Kaplan-Meier test of the mice (n = 5 per group) inoculated in the indicated cells. (F) Representative images (left panels) and histogram (right panels) showing Matrigel tube formation assay with human lymphatic endothelial cells (HLECs). HLECs were cultured in a conditioned medium derived from ovarian cancer cells treated as indicated. All *in vitro* experiments were performed in at least three biological replicates. The error bars indicate the SD of the mean. Scale bars: 100 μm. Statistical significance was assessed using a two-tailed Student's *t* test. *P < 0.05, ***P < 0.001, ****P < 0.0001.

We investigated the role of ALKBH5 in LN metastasis of ovarian cancer using a popliteal lymphatic metastasis model 25. ALKBH5 was efficiently overexpressed (**Figure 2A-B**) or inhibited (**Figure S2A-B**) in A2780 and HO8910 cells. HO8910 cell lines stably overexpressing ALKBH5 (ALK) and control cells (NC) were established and inoculated into the footpads of nude mice. Tumor cells spread from the footpad to the popliteal fossa, and those with high ALKBH5 expression had more metastases (**Figure 2C**). LYVE-1 and podoplanin (specific marker of the lymphatic endothelium, used to distinguish between lymphatic and blood vessels) staining intensity in the primary tumors was greater in mice overexpressing ALKBH5 than in control mice, as was LN metastases and tumor cell infiltration in the popliteal LNs (via H&E staining) (**Figure 2D**). Overexpression of ALKBH5 increased the tumor burden and resulted in a shorter survival (**Figure 2E**).

Next, we investigated the role of ALKBH5 overexpression and knockdown in tumor metastasis *in vitro*. The medium with high ALKBH5 expression strongly stimulated tubule formation (**Figure 2F**), the medium with ALKBH5 knockdown did not (**Figure S2C**), which suggested that high ALKBH5 expression promoted lymphatic tubule formation and then EOC metastasis* in vitro*. This finding indicated that overexpression of ALKBH5 promoted EOC cell migration and invasion (**Figure S3A-B**), but silencing of ALKBH5 reversed these effects (**Figure S2D-E**).

### ALKBH5 regulates m6A modifications of ITGB1 mRNA through a post-transcriptional mechanism

**Figure 3:**
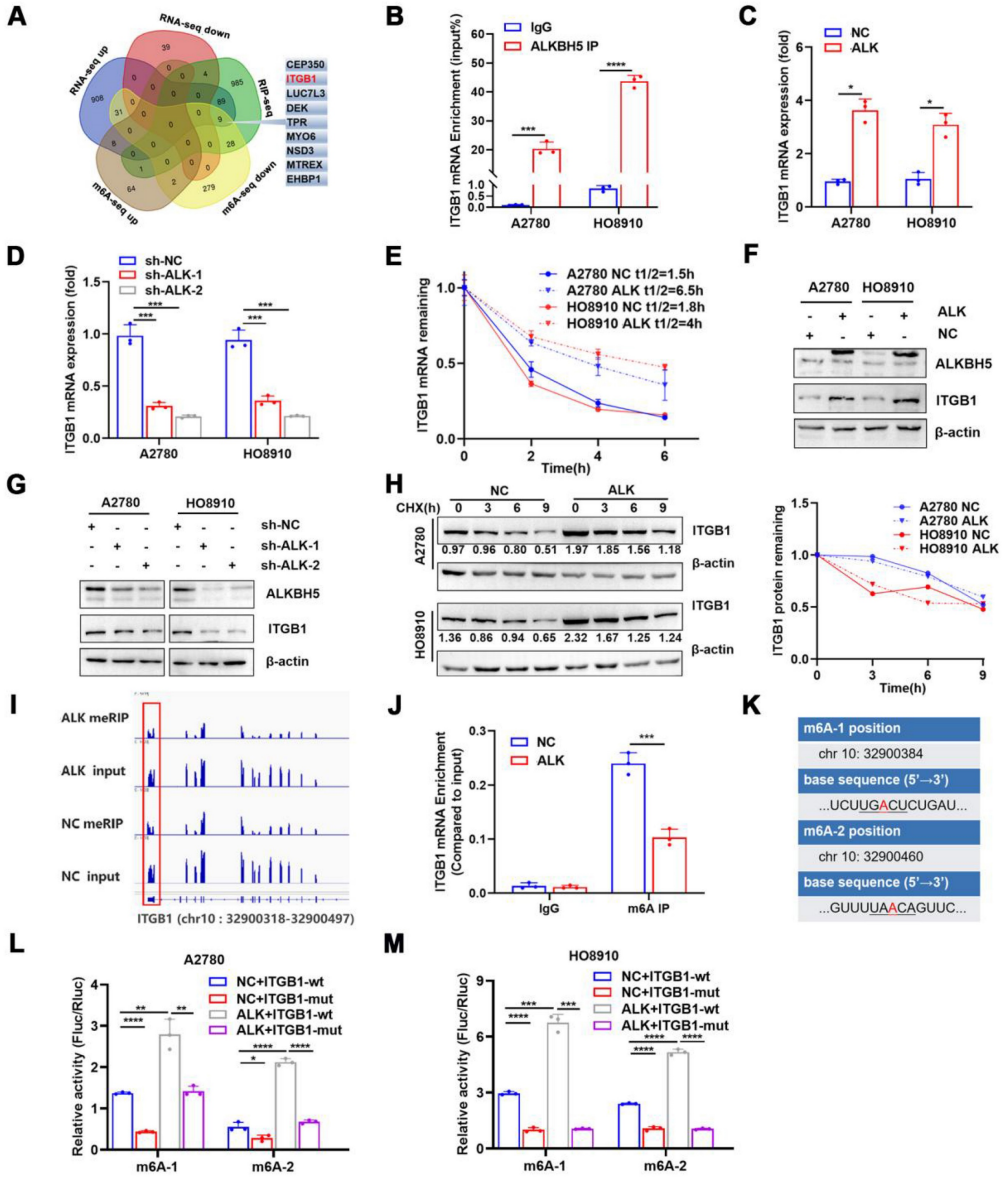
** ALKBH5 abrogates m6A modifications of ITGB1 mRNA via a post-transcriptional mechanism.** Venn diagram of genes detected using methylated RNA immunoprecipitation (MeRIP-seq), RNA-seq in the previous study, and RNA-binding protein-mRNA interactions supported by starBase data; the nine candidate target genes of ALKBH5 are shown on the right. (B) RIP-qPCR confirmed ALKBH5 binding to *ITGB1* mRNA. (C) qPCR analysis showed that ITGB1 is upregulated in EOC cells with ALKBH5 overexpression. (D) qPCR showed that ITGB1 expression was reduced upon knocking down ALKBH5 in A2780 and HO8910 cells. (E) RNA stability assay of ALKBH5-overexpressing and NC cells treated with actinomycin D for the indicated times, and qPCR analysis of mRNA expression of ITGB1. (F) Western blot analysis showed that ITGB1 was upregulated in EOC cells with ALKBH5 overexpression. (G) Western blot analysis showed that ITGB1 expression was reduced upon knocking down ALKBH5 in EOC cells. (H) Protein stability assay of ALKBH5-overexpressing and NC cells treated with cycloheximide (CHX) for the indicated times; protein expression of ITGB1 was analysed using western blot analysis (left) and quantitatively analysed (right). (I) IGV showed m6A abundance in ITGB1 mRNA transcripts in cells with ALKBH5 overexpression (MeRIP and input) and in the negative control (MeRIP and input). m6A regulation was calculated as the ratio of m6A abundance of MeRIP to input (log2 fold-change = -1.45, *P* < 0.01); (J) MeRIP-qPCR confirmed that ALKBH5 overexpression downregulated the m6A peak in the 3′ untranslated region (UTR) of *ITGB1* mRNA. (K) mRNA sequences of m6A peaks. (L and M) Relative luciferase activity of wild-type or mutant ITGB1 3′UTR luciferase reporter in A2780 (L) and HO8910 (M) with ALKBH5 overexpression and in the negative control. Error bars indicate the SD of the mean. Statistical significance was assessed using a two-tailed Student's *t* test. *P < 0.05, **P < 0.01, ***P < 0.001, ****P < 0.0001.

ALKBH5 demethylated mRNAs to promote or suppress proliferation in many cancers 26,27. m6A dot blot assays revealed that the upregulation of ALKBH5 in EOC cells reduced the m6A modification level of mRNA **(Figure S4A)**, whereas downregulation of ALKBH5 had the opposite effect **(Figure S4B)**. We previously reported alterations in m6A modifications and transcriptome in ALKBH5-overexpressing EOC 28. RNA-Seq and MeRIP-Seq were combined to identify ALKBH5 target genes, focusing on genes with low m6A levels and significant changes in mRNA expression. Concurrently, by combining protein-RNA interactions in starBase databases supported by large-scale CLIP-Seq data 29, we identified nine target genes of ALKBH5 (**Figure 3A and Table 1**). Among them, *ITGB1*, *CEP350*, *TPR*, and *EHBP1* showed a significant relationship with ALKBH5 in Gene Expression Profiling Interactive Analysis (**Figure S5A**). According to a survival study using TCGA and the Gene Expression Omnibus databases, ITGB1 and EHBP1 were both risk factors for poor prognosis (**Figure S5B**). Notably, correlation analysis revealed that ITGB1 and ALKBH5 had a substantial positive co-expression (**Figure S5C**). Therefore, ITGB1 might be an important downstream target regulated by ALKBH5.

ITGB1was an integrin family member involved in embryonic development, tumor metastasis, and angiogenesis 30,31. To determine whether the ALKBH5 protein regulates *ITGB1* mRNA expression, we firstly examined whether these molecules bind to each other. The ability of ALKBH5 to bind *ITGB1* mRNA was validated using RIP-qPCR (**Figure 3B**). Overexpression of ALKBH5 promoted *ITGB1* mRNA expression (**Figure 3C**), whereas knockdown of ALKBH5 inhibited *ITGB1* mRNA expression (**Figure 3D**). m6A modification could influence RNA metabolism, including RNA stability and translation efficiency; however, RNA stability tests revealed that *ITGB1* mRNA expression was quite stable in EOC cells overexpressing ALKBH5 (**Figure 3E**). The regulatory relationship between ALKBH5 and ITGB1 protein expression was validated via western blotting (**Figure 3F-G**). Protein stability assays revealed that overexpression of ALKBH5 did not influence the protein stability of ITGB1 (**Figure 3H**). These results suggested that ALKBH5 regulated ITGB1 protein and mRNA expression via a post-transcriptional mechanism.

We concentrated on the precise m6A sites regulated by ALKBH5 because ALKBH5 could regulate RNA metabolism by mediating m6A demethylation in *ITGB1* mRNA. According to our recent report 34, when ALKBH5 was overexpressed in A2780 cells, m6A abundance in the 3′UTR region of ITGB1 mRNA would be significantly reduced (log2 fold-change = -1.45, *P* < 0.01) (**Figure 3I**). We performed MeRIP-qPCR to confirm whether ALKBH5 overexpression reduced m6A modification levels in the 3′UTR region (chr 10:32900318-32900497) of *ITGB1* mRNA (**Figure 3J**). The distribution of m6A in RNA typically followed the DRACH (D = G/A/U, R = A/G, H = not G) motif sequence 35, 36. We identified two sites matching the DRACH sequence rules among the differential m6A modification sequences in the 3′UTR (chr 10:32900318 - 32900497) of ITGB1 (**Figure 3K**). Considered as potential m6A modification sites, the two sites were individually mutated to establish wild-type and mutant (chr 10:32900384, 32900460) luciferase reporter gene vectors. Luciferase reporter assay demonstrated that base mutation remarkably reduced the luciferin activity in cells. In contrast, enhanced expression of ALKBH5 protein increased the overall intracellular fluorescein activity (**Figure 3L-M**). These results indicated that ALKBH5 regulated *ITGB1* mRNA expression and confirmed the regulatory sites involved.

### ALKBH5 induces lymphatic metastasis in EOC by regulating ITGB1 expression

ITGB1 was thought to control tumor cell metastasis; however, its role in ovarian cancer cell migration was unclear. In ovarian cancer cells, we used two distinct siRNAs to knock down ITGB1 expression. Silencing the intracellular ITGB1 mRNA and protein expression (**Figure S6A - B**) reduced intracellular ITGB1 expression, which was associated with reduced HLEC tube formation (**Figure S6C**), tumor cell migration (**Figure S6D**), and invasion (**Figure S6E**).

**Figure 4:**
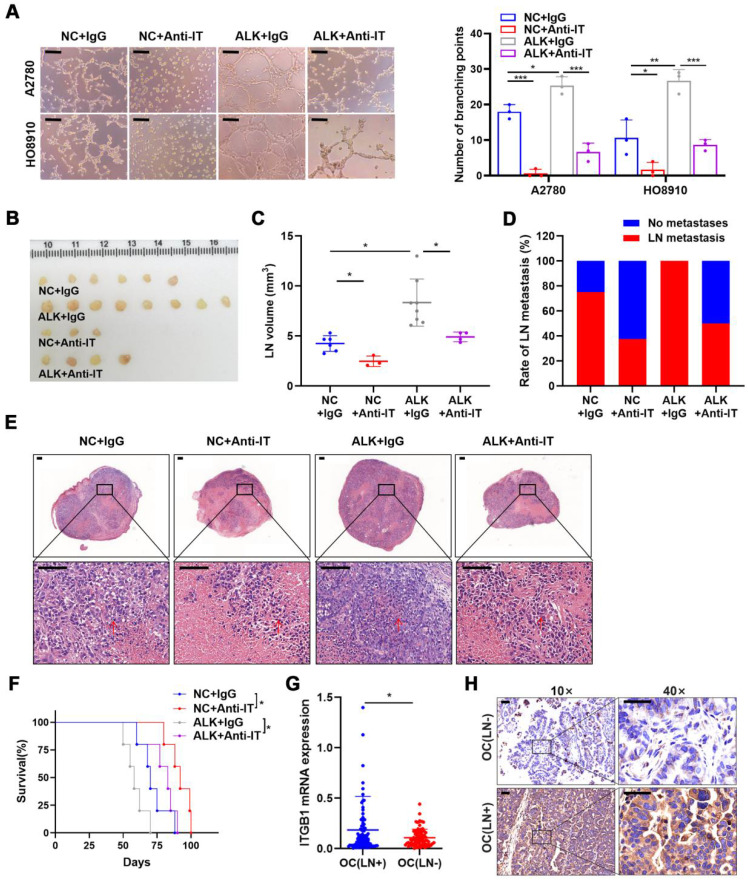
** ALKBH5 exerts lymph node (LN) metastasis in epithelial ovarian cancer by regulating ITGB1 expression.** (A) Representative images (left panels) and histogram (right panels) of Matrigel tube formation assay with human lymphatic endothelial cells (HLECs). (B) Representative images of enucleated popliteal LNs (n = 8 per group); (C) Volume of enucleated popliteal LNs (n = 8 per group). (D) Histogram of the LN metastasis ratio (n = 8 per group). (E) Representative images of haematoxylin and eosin (H&E) staining of LNs indicating tumor cell infiltration. The indicated HO8910 cells were injected into the footpads of nude mice randomized to receive either an ITGB1-blocking antibody or IgG isotype antibody. Popliteal LNs were enucleated and analysed. H&E staining of the LN status indicating tumor cell infiltration (red arrow). (F) Kaplan-Meier test of the mice (n = 5 per group) inoculated with the indicated cells. (G) qPCR analysis of *ITGB1* mRNA expression in epithelial ovarian cancer (EOC) tissues with or without LN metastasis (n = 192). (H) Immunohistochemical staining to determine ITGB1 expression in EOC tissues with or without LN metastasis. All *in vitro* experiments were performed in at least three biological replicates. Scale bars: 100 μm. Statistical significance was assessed using two-tailed Student's *t* test. *P < 0.05, **P < 0.01, ***P < 0.001, ****P < 0.0001.

Although ALKBH5 overexpression promoted LN metastasis, ITGB1 knockdown reversed the effects of ALKBH5 overexpression on lymphangiogenesis (**Figure S7A**), EOC cell migration (**Figure S7B**), and invasion (**Figure S7C**). As ITGB1 localized to the surface of the cell membrane, we introduced an ITGB1-blocking antibody to block its function and to evaluate whether the anti-ITGB1 antibody could help prevent EOC metastasis. The ITGB1-blocking antibody reduced the tumor metastasis caused by ALKBH5 overexpression and diminished lymphatic tube formation (**Figure 4A**), EOC cell migration (**Figure S8A**), and invasion (**Figure S8B**).

Moreover, considering using an ITGB1-blocking antibody in a mouse model as a new therapeutic strategy 22,23, the therapeutic effect of targeting ITGB1 was assessed in an LN metastasis model. Treatment with an ITGB1-blocking antibody led to a significant reduction of LN volume (**Figure 4B, C**) and metastasis rate (**Figure 4B-D**). H&E staining showed that ITGB1-blocking antibody treatment resulted in less tumor cell infiltration in popliteal LNs (**Figure 4E**). The decrease in tumor burden in the LNs prolonged the survival of tumor-bearing nude mice (**Figure 4F**).

Notably, ITGB1 mRNA and protein had a higher-level expression in the EOC tissues with LN metastasis than in those without LN metastasis (**Figure 4G, H**). Thus, ITGB1 was a promising target for treating patients with EOC with LN metastases.

### YTHDF2 regulates ITGB1 mRNA decay and inhibits lymphatic tube formation

**Figure 5:**
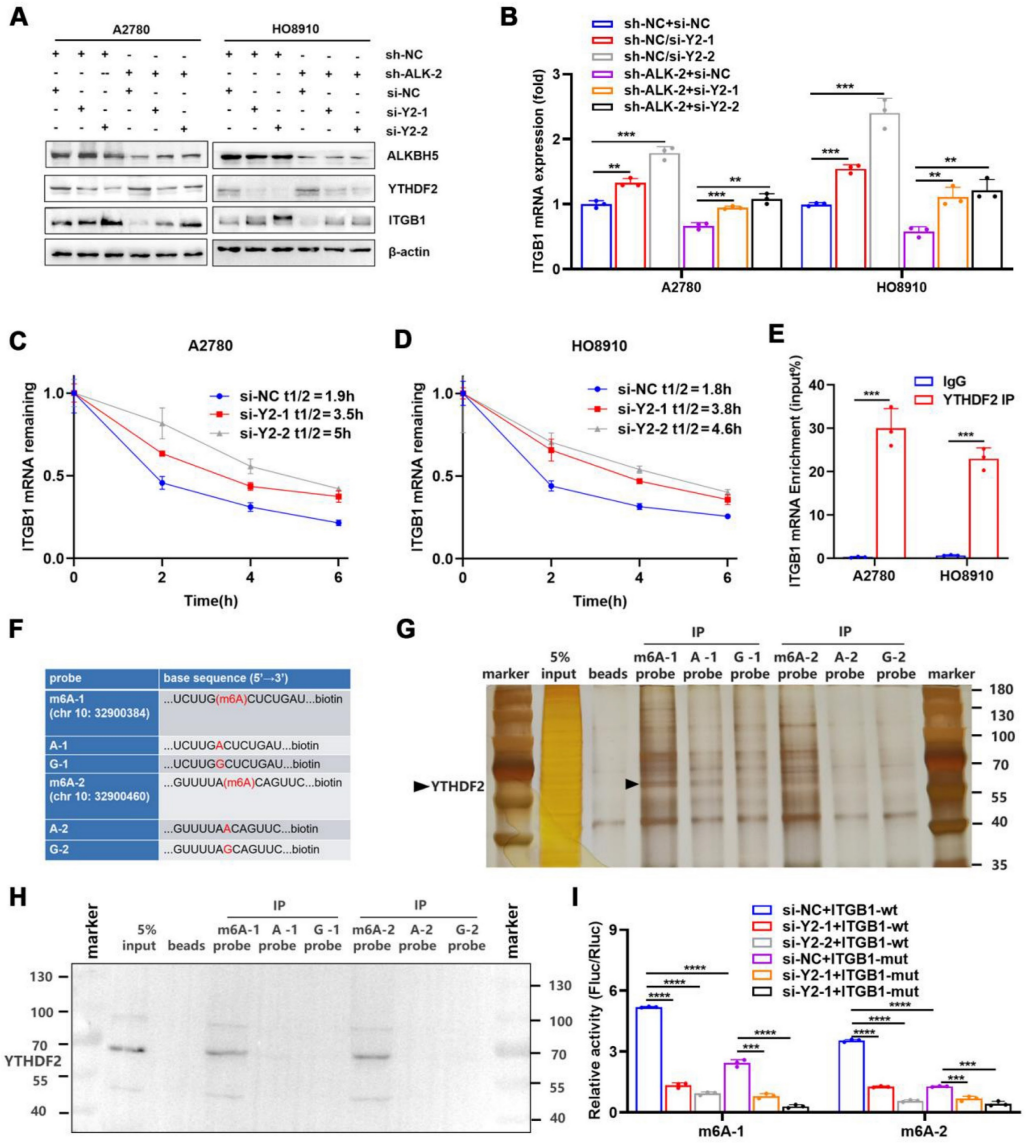
** YTHDF2 recognizes and binds *ITGB1* mRNA in an m6A-dependent manner.** (A and B) Western blot analysis (A) and qPCR (B) showed that YTHDF2 regulates ITGB1 expression in A2780 and HO8910 cells. (C and D) Increased lifespan of *ITGB1* mRNA after YTHDF2 silencing in A2780 (C) and HO8910 cells (D). (E) RIP-qPCR confirmed YTHDF2 binding to *ITGB1* mRNA. (F) RNA probe sequences for RNA pulldown. (G and H) RNA pulldown assays showed that YTHDF2 recognizes the m6A site in the 3′ untranslated region (UTR) of *ITGB1* mRNA displayed using silver staining (G) and western blotting (H). (I) Relative luciferase activity of wild-type or mutant ITGB1 3′UTR luciferase reporter in A2780 cells with YTHDF2 knockdown and the negative control. Error bars indicate mean ± SD, *P < 0.05, **P < 0.01, ***P < 0.001, ****P < 0.0001.

By recognising m6A modifications, m6A reader proteins regulated the metabolism of RNA molecules 37. We found that the abundance of m6A in *ITGB1* mRNA was reduced in cells overexpressing ALKBH5, which was accompanied by decreased mRNA stability, suggesting that YTHDF2 was involved in the recognition and regulation of m6A modifications in *ITGB1* mRNA. We used siRNA to knock down YTHDF2 to determine whether this protein regulates the expression of ITGB1. The protein (**Figure 5A**) and mRNA expression (**Figure 5B**) of ITGB1 was remarkably reduced after YTHDF2 was silenced, and this phenomenon was reversed by silencing of YTHDF2 (**Figure 5A, B**). After silencing of YTHDF2 expression in cells, the mRNA stability of ITGB1 was remarkably reduced (**Figure 5C, D**), indicating that YTHDF2 was involved in recognising the ALKBH5-regulated m6A modifications. Functionally, YTHDF2 knockdown partially rescued the LN metastasis caused by ALKBH5 downregulation during HLEC tube formation (**Figure S9A**), migration (**Figure S9B**), and invasion (**Figure S9C**).

RIP-qPCR was used to investigate whether YTHDF2 directly recognised m6A modifications in *ITGB1* mRNA and triggered RNA degradation. As expected, *ITGB1* mRNA was formed in response to YTHDF2 expression (**Figure 5E**). Furthermore, we synthetized biotinylated probes containing two sites (chr 10:32900384, 32900460) in the 3′UTR of *ITGB1* mRNA (**Figure 5F**). RNA-pulldown confirmed that the YTHDF2 was associated with m6A modification and that the m6A probe had a higher binding capacity than probe A and probe G (**Figure 5G, H**). These findings indicated that YTHDF2 could directly bind to the *ITGB1* mRNA and recognise m6A modifications in this region. To confirm the function of this interaction, we employed reporter gene vectors containing 3′UTR of wild-type or mutant *ITGB1* mRNA. As expected, the luciferase activity of the wild-type plasmid was significantly reduced after silencing YTHDF2. These results indicated that YTHDF2 recognised the m6A modifications in *ITGB1* and induced mRNA degradation (**Figure 5I**).

### ITGB1 expression enhances the phosphorylation of FAK and Src kinase to promote LN metastasis

**Figure 6:**
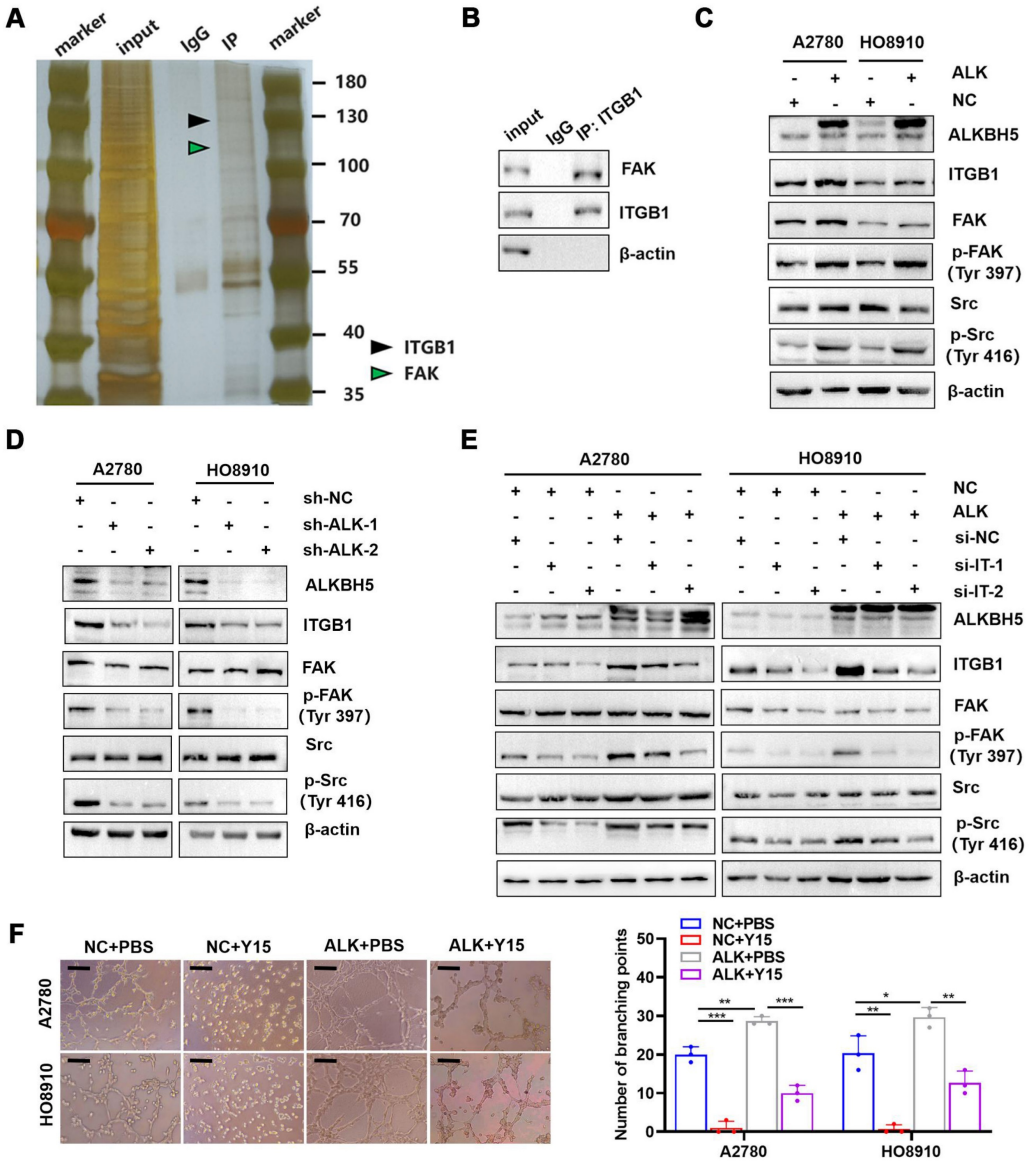
** ITGB1 regulates FAK and Src to promote lymph node (LN) metastasis in epithelial ovarian cancer.** (A and B) Co-immunoprecipitation assays showing that ITGB1 co-immunoprecipitated with FAK, as assessed via silver staining (A) and western blotting (B). (C) Upregulation of ALKBH5 promoted ITGB1 expression and phosphorylation of FAK and Src. (D) Downregulation of ALKBH5 inhibited ITGB1 expression and phosphorylation of FAK and Src. (E) ITGB1 silencing in ALKBH5-overexpressing cells reduced the phosphorylation of FAK and Src. (F) Representative images (left panels) and histogram (right panels) of Matrigel tube formation assay with human lymphatic endothelial cells (HLECs). HLECs were cultured in a conditioned medium derived from ovarian cancer cells treated as shown; all *in vitro* experiments were performed in at least three biological replicates. The error bars indicate the SD of the mean. Scale bars: 100 μm. Statistical significance was assessed using two-tailed Student's *t* test. *P < 0.05, **P < 0.01, ***P < 0.001, ****P < 0.0001.

ITGB1 aggregation induced autophosphorylation of FAK at Tyr397, which triggered downstream Src kinase phosphorylation at Tyr416, initiating a variety of molecular signals associated with tumor progression 32. Therefore, we hypothesised that ITGB1 also induced LN metastasis in ovarian cancer through a similar mechanism. Co-immunoprecipitation assays confirmed that ITGB1 bound to FAK (**Figure 6A-B**). Moreover, ITGB1 expression increased in ALKBH5-overexpressing cells, similar to FAK protein phosphorylation at position 397 and Src protein phosphorylation at Y416; however, total protein expression remained unchanged (**Figure 6C**). The opposite result was observed in cells with silenced ALKBH5 expression (**Figure 6D**). Furthermore, ITGB1 knockdown reduced FAK and Src protein phosphorylation in ALKBH5-overexpressing cells (**Figure 6E**). These findings indicated that ALKBH5 interacted with ITGB1 to increase FAK and Src phosphorylation.

Y15 was a small chemical FAK protein kinase inhibitor that suppressed the autophosphorylation activity at Tyr397 33, inhibited tumor proliferation *in vivo* and *in vitro* 34. ALKBH5-overexpressing cells were treated with Y15 to evaluate the efficacy of FAK as an intervention target against LN metastasis. Y15 blocked ALKBH5 expression by increasing lymphangiogenesis (**Figure 6F**), tumor cell migration (**Figure S10A**), and invasion (**Figure S10B**).

### Hypoxia regulates ALKBH5 expression via HIF-1α and promotes LN metastasis in EOC

**Figure 7:**
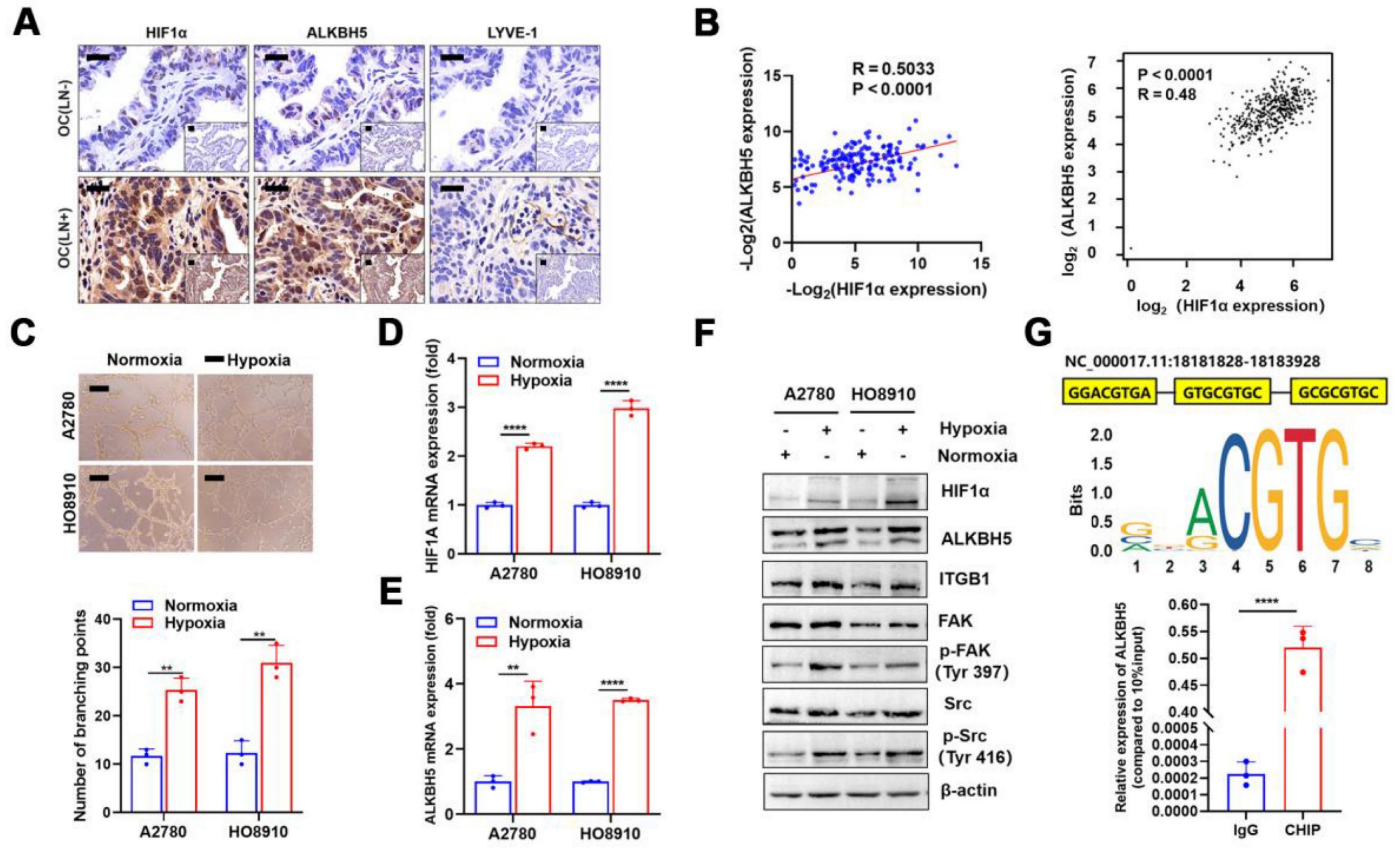
** Hypoxia regulates the expression of ALKBH5 via HIF-1α and promotes lymph node (LN) in epithelial ovarian cancer (EOC).** Immunohistochemical (IHC) staining of HIF-1α, ALKBH5, and LYVE-1 expression in EOC tissues with or without LN metastasis. (B) Correlation analysis showed that ALKBH5 and HIF-1α mRNA expression was positively correlated in our cohort (left panels) and The Cancer Genome Atlas (TCGA) (right panels). (C) Representative images (upper panels) and histogram (lower panels) of Matrigel tube formation assay with human lymphatic endothelial cells (HLECs). HLECs were cultured in a conditioned medium derived from ovarian cancer cells treated as indicated. (D and E) qPCR to examine the effect of hypoxia on the expression of HIF-1α (D) and ALKBH5 (E). (F) Western blot analysis to examine the effect of hypoxia on the protein expression of HIF-1α, ALKBH5, ITGB1, p-FAK, and p-Src. (G) Based on the transcription factor binding motif of HIF-1α predicted using the JASPAR database (upper panels), the chromatin immunoprecipitation (ChIP)-qPCR assay suggested that the ALKBH5 promoter fragment was enriched in HIF-1α (lower panels). All *in vitro* experiments were performed in at least three biological replicates. Scale bars: 100 μm. Statistical significance was assessed using a two-tailed Student's *t* test. *P < 0.05, **P < 0.01, ***P < 0.001, ****P < 0.0001.

Although we confirmed that a high ALKBH5 expression promoted EOC migration, the cause of its increased expression was unclear. The local tumor microenvironment as well as transcription factor HOXA10 could stimulate the expression of ALKBH5 in tumor cells 27,28. Recent research indicated that local hypoxia in tumor was important for EOC cell metastasis 35. Thus, we hypothesised that hypoxia-induced enhanced HIF-1α secretion also stimulated ALKBH5 expression and accelerated early lymphangiogenesis in EOC cells, thereby enhancing tumor metastasis. To evaluate this hypothesis, we performed immunohistochemical staining of clinical samples from patients with EOC with LN metastases. The expression of HIF-1α, ALKBH5, and lymphatic density (LYVE-1 marker) was significantly elevated in tumor tissues (**Figure 7A**). We also conducted qPCR to determine *ALKBH5* and *HIF-1α* mRNA expression levels in tumor samples. The expression of these two genes showed a positive correlation in correlation analysis (**Figure 7B, left**), which was confirmed through online data analysis of ovarian cancer sequencing data from TCGA (**Figure 7B, right**). Culturing EOC cells under hypoxic conditions enhanced LN metastasis, including lymphatic vessel formation (**Figure 7C**), migration (**Figure S11A**), and invasion (**Figure S11B**). These findings indicated that hypoxia could enhance ALKBH5 expression by inducing HIF-1α expression. Additionally, hypoxia significantly enhanced HIF-1α and ALKBH5 expression in tumor cells. Increased expression of downstream ITGB1, p-FAK, and p-Src was also observed (**Figure 7D-F**).

HIF-1α was primarily a transcription factor that promoted downstream gene transcription. We referred to the JASPAR database to evaluate whether the ALKBH5 promoter region contains an HIF-1α protein-binding sequence. In the ALKBH5 promoter region, we identified a motif sequence capable of binding to HIF-1α (**Figure 7G, upper**). ChIP-qPCR also confirmed that HIF-1α bound to the ALKBH5 promoter region after hypoxia exposure (**Figure 7G, lower**).

## Discussion

LN metastasis is a poor prognostic factor in several malignancies. Tumor cells must overcome multiple barriers to migrate into LNs, including the formation of lymphatic vessels, which are the main route tumor cells spread to regional LNs. Tumor-neoplastic lymphatic vessels may be more important in tumor migration than lymphatic veins around the tumor. VEGF-C, VEGF-D, and VEGFR-3 expression is crucial for the formation of new lymphatic capillaries in many types of tumors. In an ovarian cancer xenograft model, Du *et al.* 36 found that VEGF-D induced the expansion of draining lymphatic vessels and that tumor lymphangiogenesis increased LN metastasis. Matrix protein (MP)^37^ and secreted protein acidic and rich in cysteine (SPARC) 38 effectively inhibited lymphangiogenesis in ovarian cancer by reducing VEGF-D expression. The molecular mechanisms that m6A-related enzyme regulated lymphatic capillary formation were recently determined. According to Yu et al., IGF2BP2 promoted lymphangiogenesis in head and neck squamous carcinoma cells by stabilising Slug mRNA in an m6A-dependent manner 19. Similarly, a study of diabetes showed that methyltransferase improves wound healing in diabetic foot ulcers by boosting the m6A modification of VEGF-C mRNA, thereby enhancing VEGFR3-mediated lymphangiogenesis 39. We found that EOC cells accelerated lymphangiogenesis and LN metastasis by expressing the demethylase ALKBH5. In addition, ALKBH5 regulates ITGB1 expression through m6A demethylation, which may be a molecular mechanism for promoting EOC migration. However, considering that ALKBH5 regulates hundreds of downstream genes, we hypothesised that ALKBH5 forms a massive regulatory network via the m6A methylation regulatory mechanism and plays a role in EOC migration, which requires further investigation.

Integrins are heterodimeric cell surface adhesion molecules found in all nucleated cells. The 18 α- and eight β-subunits form 24 different heterodimers, each with functional and tissue specificity. They integrate processes in the intracellular compartment with those in the extracellular environment 40. Integrins and integrin-dependent processes are associated with almost all stages of cancer progression, including cancer initiation and proliferation, local invasion and neovascularization into the vasculature, survival of circulating tumor cells, stimulation of the metastatic niche, extravasation into the secondary site, and metastatic colonisation of new tissue 41. Among these intergrins, ITGB1 promotes tumor metastasis and progression in various cancers 42,43. Furthermore, ITGB1 is implicated in lymphatic vascular development and lymphangiogenesis under both physiological and pathological conditions, such as breast cancer 44. The enhanced ITGB1 expression in malignancies is thought to be caused by transcriptional or post-transcriptional mechanisms. Cdc42, for example, activates SRF to regulate ITGB1 expression at the transcriptional level 45. Through a post-transcriptional regulation mechanism, microRNA-29c suppresses the *ITGB1* mRNA 46.

We present a regulatory mechanism for *ITGB1* mRNA expression. The demethylase ALKBH5 and reader protein YTHDF2 regulate m6A modification in ITGB1 mRNA. Enhanced ALKBH5 expression in EOC cells may lead to a reduction in m6A modification of ITGB1 mRNA and inhibit the YTHDF2-mediated m6A-dependent degradation mechanism, thus enhancing *ITGB1* mRNA stabilisation and expression. However, we identified other chemical modifications in *ITGB1* mRNA, such as m1A and m5C, in a high-throughput database of RNA modifications, suggesting that more diverse mechanisms are involved in metabolic regulation of mRNA. In addition, integrin-targeting antibodies, such as the pan-αv antibodies abituzumab and intetumumab, have been evaluated in clinical studies of colorectal cancer and melanoma. We also observed that inhibition of ITGB1 with recombinant antibodies prevented lymphangiogenesis and tumor metastasis in EOC, indicating the potential efficacy of anti-ITGB1 therapeutics in EOC.

The non-receptor protein tyrosine kinases FAK and Src are essential for integrin-associated signal transduction 47. In many human malignancies, aberrant FAK/Src signalling leads to increased tumor migratory and invasive capabilities 47. Therefore, targeting FAK using small molecules is a promising cancer treatment. Six FAK inhibitors are being evaluated in clinical studies: GSK-2256098 (phase I), VS-6063 (phase II), CEP-37440 (phase I), VS-6062 (phase I), VS-4718 (phase I), and BI-853520 (phase I). The p-FAK inhibitor Y15 exerts anti-lymphangiogenesis and anti-metastatic effects in EOC. Thus, Y15 is a promising small molecule for treating LN metastases; however, further studies in preclinical models are needed to confirm this finding.

Tumor progression is influenced by the crosstalk between cancer cells and the microenvironment. Hypoxia, a hallmark of cancer microenvironment, plays a key role in tumor cell metastasis 48,49. In addition, cellular responses to external stimulation and stress are mediated by m6A. According to Wang *et al.* 50, hypoxia systematically reprograms the m6A epitranscriptome, including a reduction in total m6A levels in RNA, widespread downregulation of m6A readers, and systematic alteration of m6A levels in numerous transcripts. Hypoxia induces the breast cancer stem cell phenotype through HIF-1α-dependent and ALKBH5-mediated m6A-demethylation of *NANOG* mRNA 35. We find that hypoxia-induced HIF-1α activates the ALKBH5 axis and accelerates tumor metastasis. Thus, targeting hypoxia and its associated pathways could be a promising therapeutic strategy against tumor metastasis.

## Conclusion

Hypoxia promotes the expression of HIF-1α and ALKBH5; ALKBH5 regulates the expression of ITGB1 in an m6A-YTHDF2-dependent manner, triggers the phosphorylation of FAK and Src, and promotes EOC cancer lymphangiogenesis and metastasis.

## Supplementary Material

Supplementary figures and tables.

## Figures and Tables

**Table 1:** List of nine genes selected by MeRIP-seq, RNA-seq and starbase data.

	MeRIP-seq		RNA-seq		m6A peak location		
Gene Name	*P*	log_2_fc	*P*	log_2_fc	Seq names	start	end
ITGB1	0.00	-1.45	0.01	2.05	chr10	32900318	32900497
MTREX	0.00	-1.06	0.01	2.07	chr5	55307989	55322407
NSD3	0.00	-1.20	0.01	2.07	chr8	38329492	38331568
EHBP1	0.00	-1.99	0.00	2.16	chr2	62706724	62707226
LUC7L3	0.00	-1.17	0.00	2.62	chr17	50752211	50752660
DEK	0.00	-1.04	0.00	2.64	chr6	18236465	18237214
MYO6	0.00	-1.06	0.00	3.23	chr6	75886963	75894840
TPR	0.00	-1.26	0.00	4.02	chr1	186325762	186327505
CEP350	0.00	-1.77	0.00	4.21	chr1	180094282	180095825

## Abbreviations

3'-UTR: three prime untranslated region
5'-UTR: five prime untranslated region
Act-D: Actinomycin-D
ALKBH5: AlkB homolog 5
ChIP: chromatin immunoprecipitation
CHX: cycloheximide
CO-IP: co-immunoprecipitation
EOC: epithelial ovarian cancer
FAK: focal adhesion kinase
FIGO: Federation Internationale of Gynecologie And Obstetrigue
FTO: fat mass and obesity associated
HIF-1α: hypoxia inducible factor 1 subunit alpha
HLEC: human lymphatic endothelial cell
HOXA10: Homeobox A10
IF: immunofluorescence
IHC: immunohistochemistry
IGF2BP: insulin like growth factor 2 mRNA binding protein
ITGB1: integrin subunit beta 1
LN: lymph node
m6A: N6-methyladenosinemethyltransferase-like
METTL3: methyltransferase-like 3
METTL14: methyltransferase-like 14
MeRIP-seq: m6A-modified RNA immunoprecipitation sequencing
mRNA: messenger RNA
mRNA-seq: mRNA sequencing
Mut: mutated-type
OC: ovarian cancer
OS: overall survival
PBS: phosphate buffer saline
qRT-PCR: qualified Real-time PCR
RIP: RNA binding protein immunoprecipitation
Src: Src proto-oncogene
TCGA: The Cancer Genome Atlas
TF: transcription factor
VEGF: vascular endothelial growth factor
VEGFR: vascular endothelial growth factor receptor
WT: wide-type
WTAP: WT1 associated protein
YTHDF: YTH domain family
