# Supplementary material for: ALKBH5 activates FAK signaling through m6A demethylation in ITGB1 mRNA and enhances tumor-associated lymphangiogenesis and lymph node metastasis in ovarian cancer
Source: Theranostics. 2023 Jan 5;13(2):833–48. doi: 10.7150/thno.77441 (PMC9830429; doi:10.7150/thno.77441)
Supplement: Supplementary file 1 — Supplementary figures and tables. [file thnov13p0833s1.pdf]

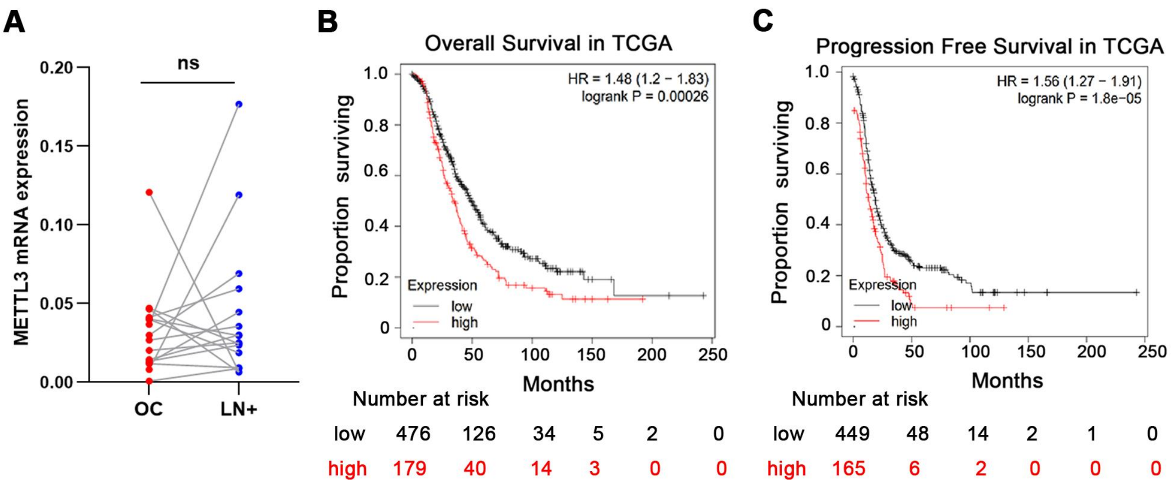

Figure S1. ***ALKBH5*** **and** ***METTL3*** **mRNA expression in patients**

(A) qPCR analysis of *ALKBH5* mRNA expression in primary tumors and lymph node (LN) metastases in the same patient (n = 16 pairs); (B) Overall survival of patients with epithelial ovarian cancer (EOC) relative to ALKBH5 expression in The Cancer Genome Atlas (TCGA) (n = 1657). (C) Progression-free survival of patients with advanced EOC relative to ALKBH5 expression in TCGA (n = 1436). ns denotes not significant.

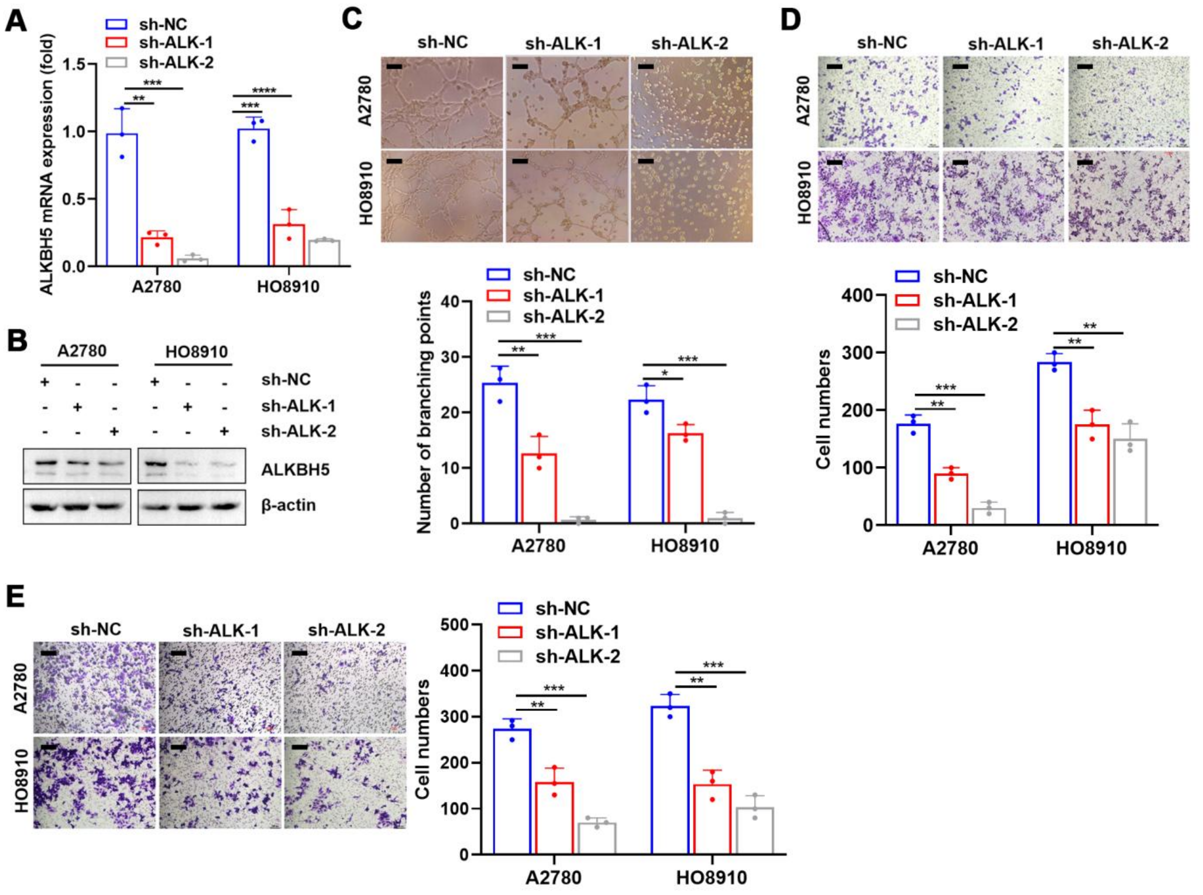

Figure S2. ALKBH5 silencing inhibits lymph node (LN) metastasis *in vitro*

(A and B) qPCR (A) and western blot (B) analysis of the knockdown efficiency of ALKBH5 in A2780 and HO8910 cells. (C) Representative images (upper panels) and histogram (lower panels) of Matrigel tube formation assay with human lymphatic endothelial cells (HLECs). HLECs were cultured in a conditioned medium derived from ovarian cancer cells treated as indicated. (D and E) Representative images (upper panels) and histogram (lower panels) of migration (D) and invasion (E) assays of A2780 and HO8910 cells; all *in vitro* experiments were performed in at least three biological replicates. Error bars indicate the SD of the mean. Scale bars: 100 μm. Statistical significance was assessed using a two-tailed Student's t test. $^{**}P < 0.01$, $^{***}P < 0.001$.

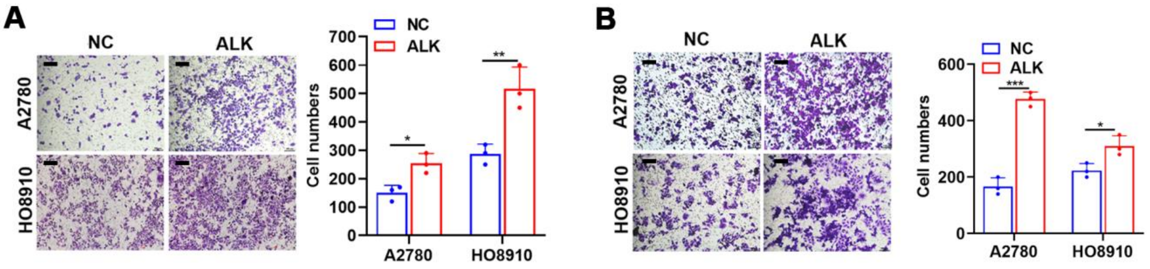

Figure S3. ALKBH5 overexpression promotes tumor cell migration and invasion *in vitro*

(A and B) Representative images (left panels) and histogram (right panels) of (A) migration and (B) invasion assay of A2780 and HO8910 cells. All *in vitro* experiments were performed in at least three biological replicates. Error bars indicate the SD of the mean. Scale bars: 100 μm. Statistical significance was assessed using a two-tailed Student's *t* test. *$P < 0.05$, **$P < 0.01$, ***$P < 0.001$.

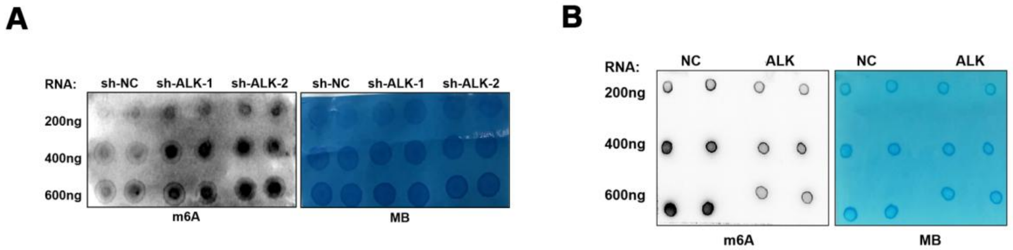

Figure S4. ALKBH5 abrogates m6A modifications in ovarian cancer cells

(A) m6A dot-blot assay showed that ALKBH5 downregulation significantly increased the m6A modification level in A2780 cells; (B) m6A dot-blot assay showed that ALKBH5 overexpression significantly reduced the m6A modification level in A2780 cells.

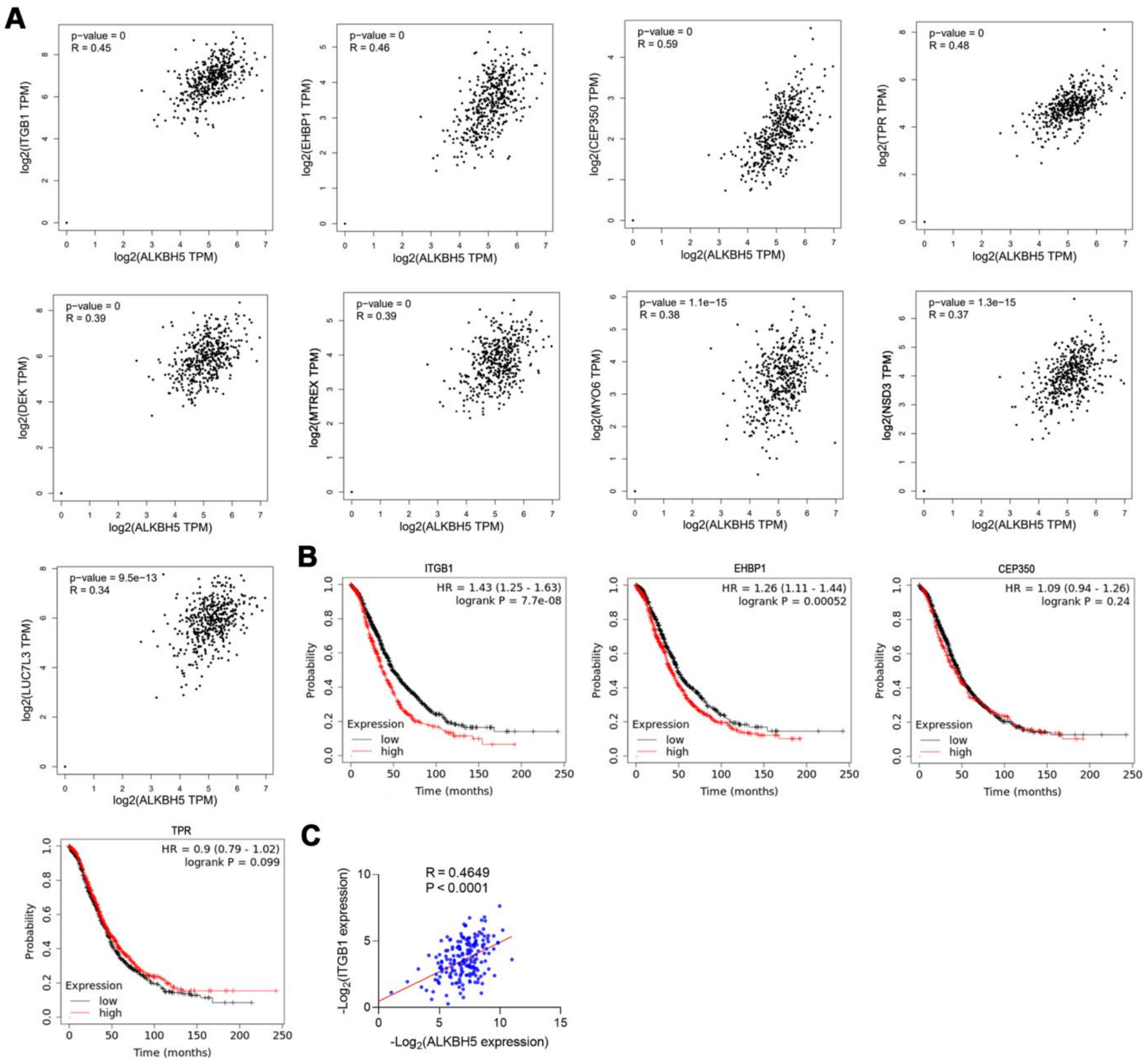

Figure S5. Expression of genes downstream of ALKBH5 in patients and patient survival

(A) Correlation analyses of ALKBH5 and expression of nine target genes in The Cancer Genome Atlas (TCGA) database. (B) Overall survival of patients with ovarian cancer in TCGA data according to the expression of the nine target genes. (C) qPCR analysis of the expression of four genes after the upregulation of ALKBH5 in A2780 cells. (D) Correlation analyses of ALKBH5 and ITGB1 expression in our data (n = 192 cases).

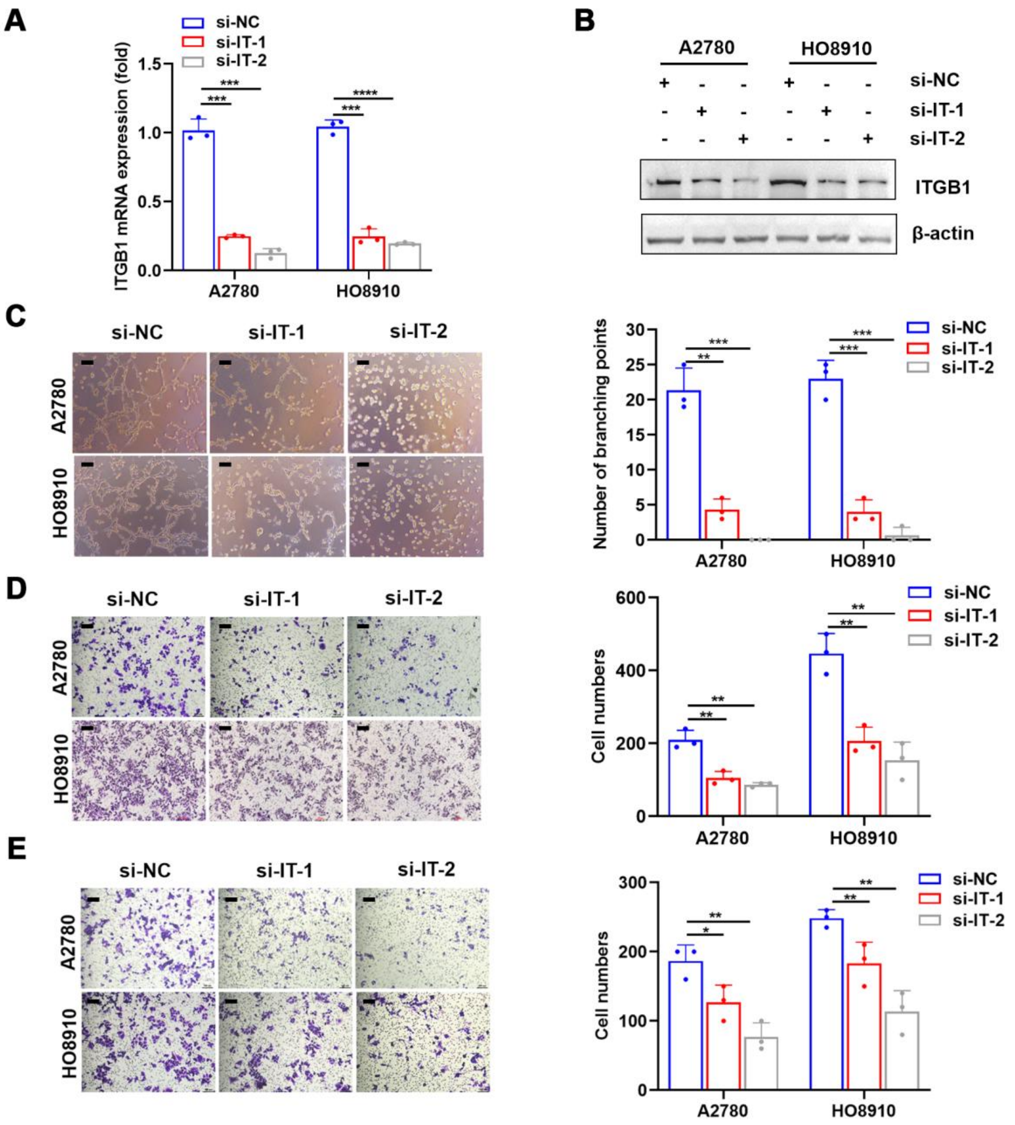

Figure S6. ITGB1 silencing inhibits lymph node (LN) metastasis *in vitro*

(A and B) qPCR and western blot (B) analysis of the transfection efficiency of downregulated-ITGB1 siRNA in A2780 and HO8910 cells. (C) Representative images (left panels) and histogram (right panels) of Matrigel tube formation assay with human lymphatic endothelial cells (HLECs). HLECs were cultured in a conditioned medium derived from ovarian cancer cells treated as indicated. (D and E) Representative images (left panels) and histogram (right panels) of migration (D) and invasion (E) assay with A2780 and HO8910 cells. All *in vitro* experiments were performed in at least three biological replicates. The error bars indicate the SD of the mean. Scale bars: 100 μm. Statistical significance was assessed using a two-tailed Student's *t* test. *$P < 0.05$, **$P < 0.01$, ***$P < 0.001$, ****$P < 0.0001$.

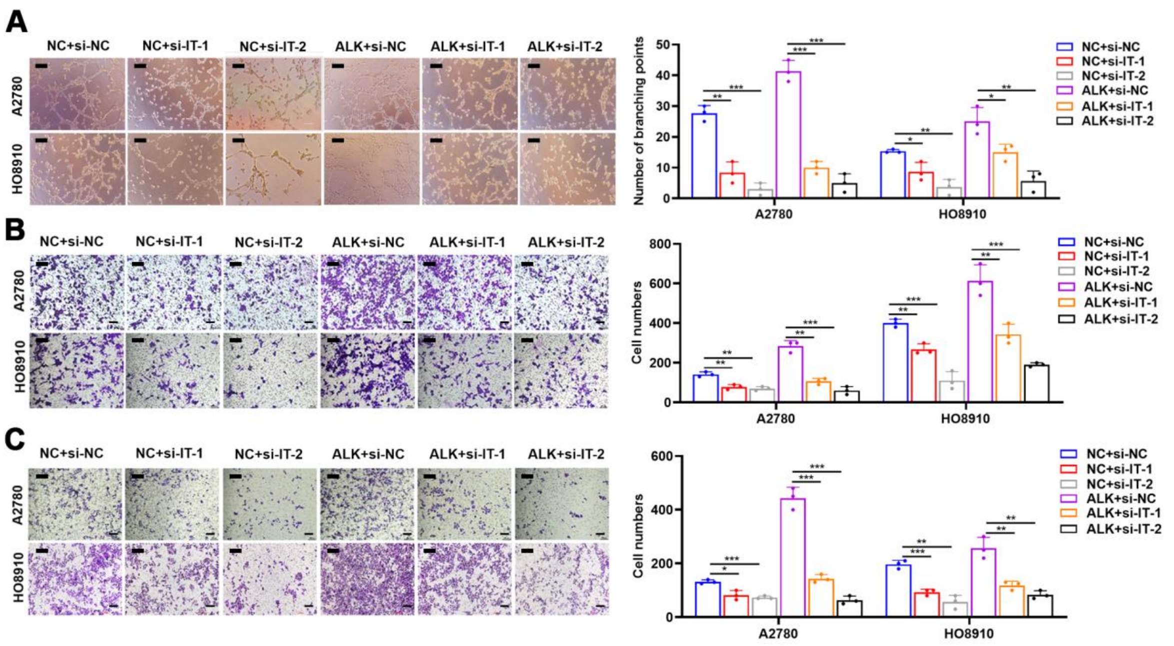

Figure S7. ITGB1 silencing rescues the ALKBH5 overexpression-induced lymph node (LN) metastasis *in vitro*

(A) ITGB1 silencing rescues ALKBH5 overexpression-induced lymphatic tube formation. (B and C) Specific siRNAs that knock down ITGB1 expression in EOC cells with ALKBH5 overexpression significantly suppressed migration (B) and invasion (C). All *in vitro* experiments were performed in at least three biological replicates. Error bars indicate the SD of the mean. Scale bars: 100 μm. Statistical significance was assessed using a two-tailed Student's *t* test. **$P < 0.01$, ***$P < 0.001$, ****$P < 0.0001$.

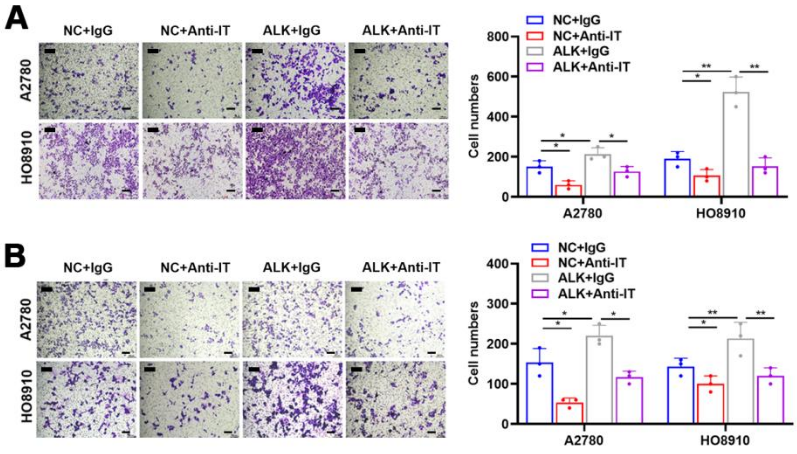

Figure S8. Blocking of ITGB1 rescues the ALKBH5 overexpression-induced tumor cell migration and invasion *in vitro*

(A) Representative images (left panels) and histogram (right panels) of the migration assay with A2780 and HO8910 cells. (B) Representative images (upper panels) and histogram (lower panels) of invasion assay with A2780 and HO8910 cells; all *in vitro* experiments were performed in at least three biological replicates. The error bars indicate the SD of the mean. Scale bars: 100 μm. Statistical significance was assessed using a two-tailed Student's *t* test. $*P < 0.05$, $**P < 0.01$.

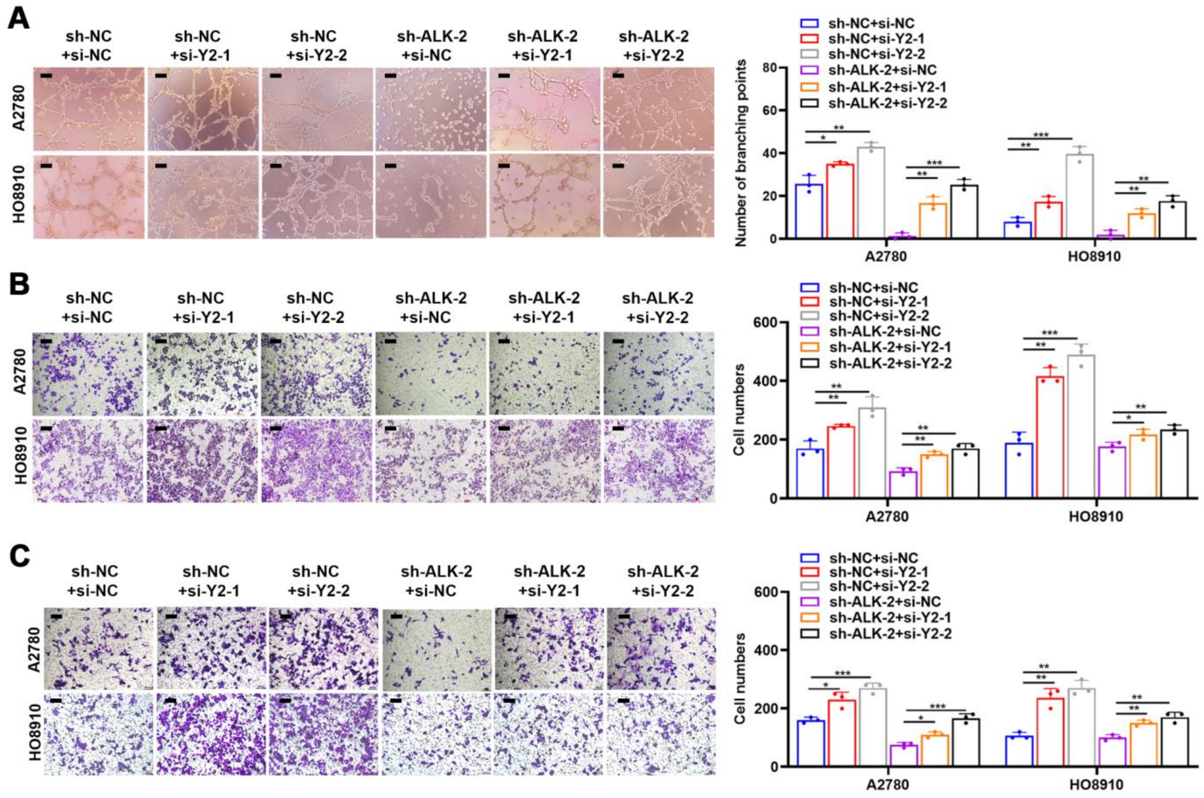

Figure S9. Knockdown of YTHDF2 partially rescues the LN metastasis caused by ALKBH5 downregulation *in vitro*

(A) Representative images (left panels) and histogram (right panels) of Matrigel tube formation assay with human lymphatic endothelial cells (HLECs). HLECs were cultured in a conditioned medium derived from ovarian cancer cells treated as indicated. (B and C) Representative images (left panels) and histogram (right panels) of migration (B) and invasion (C) assay with A2780 and HO8910 cells; all *in vitro* experiments were performed in at least three biological replicates. Error bars indicate the SD of the mean. Scale bars: 100 μm. Statistical significance was assessed using a two-tailed Student's *t* test. **P* < 0.05, ***P* < 0.01, ****P* < 0.001, *****P* < 0.0001.

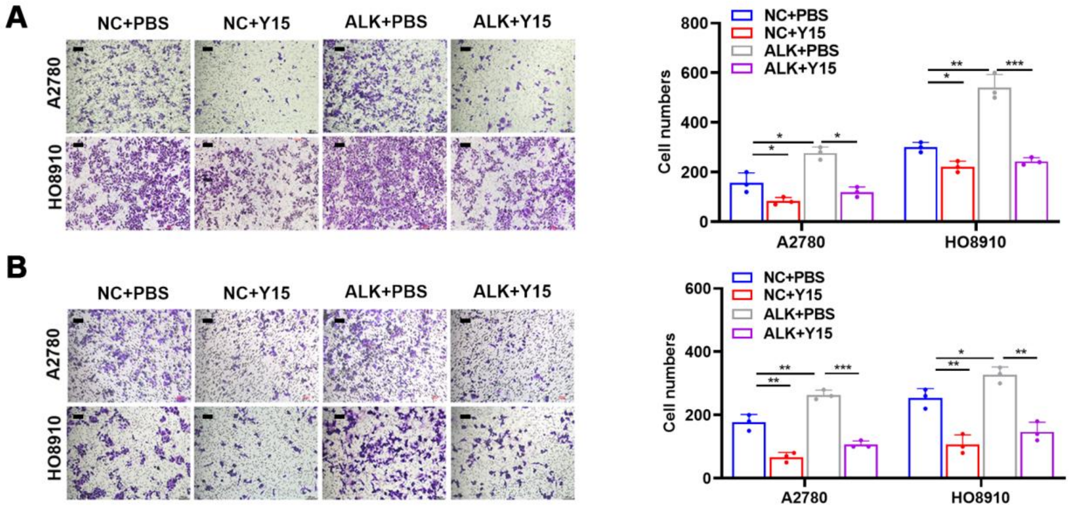

Figure S10. Y15, a p-FAK (Tyr397) inhibitor, blocks ALKBH5 expression during tumor cell migration and invasion

(A and B) Representative images (left panels) and histogram (right panels) of migration (A) and invasion (B) assay with A2780 and HO8910 cells; all *in vitro* experiments were performed in at least three biological replicates. Error bars indicate the SD of the mean. Scale bars: 100 μm. Statistical significance was assessed using a two-tailed Student's *t* test. $^*P < 0.05$, $^{**}P < 0.01$, $^{***}P < 0.001$.

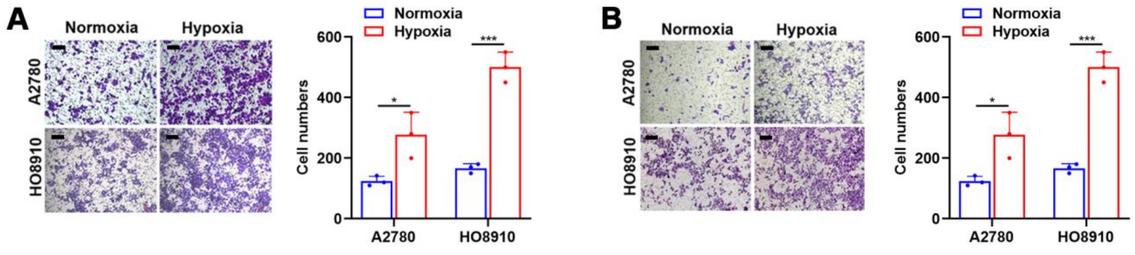

Figure S11. Hypoxia promotes tumor cell migration and invasion *in vitro*

(A and B) Representative images (left panels) and histogram (right panels) of migration (A) and invasion (B) assay with A2780 and HO8910 cells; all *in vitro* experiments were performed in at least three biological replicates. Error bars indicate the SD of the mean. Scale bars: 100 μm. Statistical significance was assessed using a two-tailed Student's *t* test. $^*P < 0.05$, $^{***}P < 0.0$

Table S1: The clinical characteristics of 192 EOC patients and the descriptive analysis of ALKBH5 mRNA expression

Characteristic	All	ALKBH5 expression		*P* value
		High-expression	Low-expression	
**Total**	192			
**Age at surgery**				0.134
**＜60**	122	56	66	
**≥60**	70	40	30	
**Histology type**				0.082
**Serous**	135	62	73	
**Other**	57	34	23	
**Grade**				0.008*
**G1+G2**	61	22	39	
**G3+G4**	131	74	57	
**FIGO Stage**				0.035*
**I+II**	33	11	22	
**III+IV**	159	85	74	
**Tumor size**				0.191
**＜10cm**	85	38	47	
**≥10cm**	107	58	49	
**Lymph node metastasis**				0.004*
**Positive**	96	58	38	
**Negative**	96	38	58	
**Peritoneal cytology**				0.559
**Positive**	110	57	53	
**Negative**	82	39	43	
**Complications**				0.209
**With diabetes**	39	23	16	
**Without diabetes**	153	73	80	

Table S2：Primers used for qPCR assay

Gene	Forward (5´-3´)	Reverse (5´-3´)
**ALKBH5**	CGGCGAAGGCTACACTTACG	CCACCAGCTTTTGGATCACCA
**FTO**	AACACCAGGCTCTTTACGGTC	TGTCCGTTGTAGGATGAACCC
**METTL3**	TTGTCTCCAACCTTCCGTAGT	CCAGATCAGAGAGGTGGTGTAG
**METTL14**	GAGTGTGTTTACGAAAATGGGGT	CCGTCTGTGCTACGCTTCA
**WTAP**	CTTCCCAAGAAGGTTCGATTGA	TCAGACTCTCTTAGGCCAGTTAC
**ZC3H13**	TCTGATAGCACATCCCGAAGA	CAGCCAGTTACGGCACTGT
**ITGB1**	CAAGAGAGCTGAAGACTATCCCA	TGAAGTCCGAAGTAATCCTCCT
**YTHDF2**	AGCCCCACTTCCTACCAGATG	TGAGAACTGTTATTTCCCCATGC
**HIF-1α**	ATCCATGTGACCATGAGGAAATG	TCGGCTAGTTAGGGTACACTTC
**β-actin**	GCTGTGCTATCCCTGTACGC	TGCCTCAGGGCAGCGGAACC
**Primers used for MeRIP-qPCR**		
**ITGB1(chr 10: 32900318-32900497)**	CCATGATTCAGAGAGCCCAA	GTCTTACTTTGAGTTAGTGCCAT
**Primers used for ChIP-qPCR**		
**ALKBH5**	CTTAGCCTTGCGCCCGTTC	AAACTTCTCAGACTGCGGGAC

Table S3：Antibodies used in this study.

Antibodies	Antibodies	Concentration
**Anti-ALKBH5**	16837-1-AP, Proteintech	1:1000 (WB), 1:400 (IHC and IF), 5 μg for RIP
**Anti-ITGB1**	ab179471, Abcame	1:2000 (WB), 1:1000 (IHC)
**Anti-ITGB1**	sc-13590L, Santa Cruz	5 μg for co-IP
**Anti-LYVE-1**	Ab219556, Abcam	1:1000 (IHC and IF)
**Anti-YTHDF2**	24744-1-AP, Proteintech	1:1000 (WB), 4 μg for RIP
**Anti-FAK**	3285T, CST	1:1000 (WB)
**Anti-p-FAK(Tyr397)**	8556T, CST	1:1000 (WB)
**Anti-p-FAK(Tyr397)**	AF3398, Affinity	1:200 (IHC)
**Anti-Src**	2109S, CST	1:1000 (WB)
**Anti-p-Src (Tyr416)**	6943S, CST	1:1000 (WB)
**Anti-HIF1α**	20960-1-AP, Proteintech	1:1000 (WB), 1:400 (IHC), 4 μg for ChIP
**Anti-Podoplanin**	Ab256561,Abcame	1:400 (IF)
**Anti-m6A**	ab232905, Abcam	1:400 (dot blot)
**Anti-m6A**	ab208577, Abcam	10 μg for MeRIP
**Anti-β-actin**	GB15003, Servicebio	1:1000 (WB)

Table S4. The target sequences of genes

gene	target sequences (5´-3´)
sh-ALKBH5-1	UCAGAUCGCCUGUCAGGAATT
sh-ALKBH5-2	GGAUAUGCUGCUGAUGAA ATT
si-ITGB1-1	AUGGGACACGGGUGAAAAUTT
si-ITGB1-2	GCUCAGUCUUACUAAUAAATT
si-YTHDF2-1	TTGGCTATGGGAACGTCTT
si-YTHDF2-2	CAAGGAAACAAAGTGCAAA

Table S5: The RNA probe sequences for RNA-pulldown

Probe	Position	Sequences(5'-3')
m6A-1 probe	Chr10: 32900384	AUCGGAUGUCUUG(m6A)CUCUGAUGUAUUUUAUCAG-biotin
G-1 probe		AUCGGAUGUCUUGGCUCUGAUGUAUUUUAUCAG-biotin
A-1 probe		AUCGGAUGUCUUGACUCUGAUGUAUUUUAUCAG-biotin
m6A-2 probe	Chr10: 32900460	GUGCCUUUAGUUUUA(m6A)CAGUUCACUUUUUACAG-biotin
G-2 probe		GUGCCUUUAGUUUUAGCAGUUCACUUUUUACAG-biotin
A-2 probe		GUGCCUUUAGUUUUAACAGUUCACUUUUUACAG-biotin

Table S6：Detailed RNA seqTences of dual-luciferase reporter

LTciferase reporter	Position	DNA seqTences
ITGB1 wt-1	Chr10: 32900384	AAAUACUUAAAUCGGAUGUCUUGACUCUGAUGUAUUUUAUCAGGUUGUGUGCAUGAAAUUUUUAUAGAUUAAAGAAGUUGAGGAAAAGCA
ITGB1 mut-1	Chr10: 32900384	AAAUACUUAAAUCGGAUGUCUUGGCUCUGAUGUAUUUUAUCAGGUUGUGUGCAUGAAAUUUUUAUAGAUUAAAGAAGUUGAGGAAAAGCA
ITGB1 wt-2	Chr10: 32900460	ACUAGUCACAUUCUUGUUUUAAGUGCCUUUAGUUUUAACAGUUCACUUUUUACAGUGCUAUUUACUGAAGUUAUUUAUUAAAUAUGCCUA
ITGB1 Mut-2	Chr10: 32900460	ACUAGUCACAUUCUUGUUUUAAGUGCCUUUAGUUUUAGCAGUUCACUUUUUACAGUGCUAUUUACUGAAGUUAUUUAUUAAAUAUGCCUA
